# Enhancing Human Action Inhibition Through Cortico–Cortical Paired Associative Stimulation

**DOI:** 10.1111/nyas.70077

**Published:** 2025-09-14

**Authors:** Lorenzo Però, Nicolò Arlati, Laura Lenzi, Thomas Quettier, Simone Battaglia, Sara Borgomaneri

**Affiliations:** ^1^ Center For Studies and Research in Cognitive Neuroscience, Department of Psychology “Renzo Canestrari,” Cesena Campus Alma Mater Studiorum Università di Bologna Cesena Italy; ^2^ Department of Psychology University of Torino Torino Italy

**Keywords:** action inhibition network, cortico–cortical paired associative stimulation, Hebbian plasticity, reactive inhibition, stop signal task

## Abstract

Reactive inhibition is crucial for preventing inappropriate actions, and impairments in this ability are common in various disorders, with the underlying neural mechanisms poorly understood. To explore the neural dynamics of such an ability, we used a novel transcranial magnetic stimulation (TMS) protocol, cortico–cortical paired associative stimulation (ccPAS), to induce Hebbian spike‐timing‐dependent plasticity (STDP). Our goal was to investigate the functional relevance of key brain regions in the action inhibition network (AIN). Healthy participants underwent ccPAS targeting functional connectivity between AIN regions: pre/supplementary motor area (preSMA/SMA) to the left motor cortex (lM1), right inferior frontal gyrus (rIFG) to lM1, or right M1 (rM1) to lM1. Participants completed a stop signal task (SST) before and after ccPAS stimulation. Motor evoked potentials (MEP) were recorded during ccPAS to assess network plasticity, and resting motor threshold (rMT) was measured for global motor excitability. Reactive inhibition improved selectively in the preSMA/SMA‐lM1 group, with MEPs increasing after preSMA/SMA‐lM1 and rM1‐lM1 stimulation, suggesting facilitatory modulations. rMT correlated with behavioral improvement in the preSMA/SMA group. These findings demonstrate that ccPAS improved reactive inhibition, enhancing plasticity between preSMA/SMA and lM1, providing insights into the AIN's functional mechanism.

## Graphical abstract

Reactive inhibition is crucial for preventing inappropriate actions. We used a TMS protocol and cortico–cortical paired associative stimulation (ccPAS). Participants underwent ccPAS targeting functional connectivity between action inhibition network regions and completed a stop signal task before and after ccPAS stimulation. Reactive inhibition improved in the preSMA/SMA‐lM1 group, with motor evoked potentials increasing after preSMA/SMA‐lM1 and rM1‐lM1 stimulation. ccPAS also enhanced plasticity between preSMA/SMA and lM1.

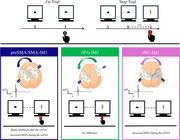

## 1 Introduction

Successful action inhibition represents a crucial ability in a complex environment. Such ability is supported by a series of brain regions, part of the so‐called action inhibition network (AIN; see [1] for a meta‐analysis and [2] for an updated activation likelihood estimation [ALE] meta‐analysis). However, the exact role of each area of the AIN network and how each area interacts with the primary motor cortex (M1) to suppress an ongoing action is still obscure. Transcranial magnetic stimulation (TMS) studies started to investigate the neural bases of action inhibition almost 20 years ago (for a review on the topic, see [3]) and, although sometimes controversial, results suggest that both the presupplementary motor cortex (preSMA) and the inferior frontal gyrus (IFG; especially the right) may play a crucial role in action inhibition. In our recent TMS study [4], we moved a step forward by investigating whether specific neural circuits engaged by action suppression are differently modulated when emotional signals are used as stop signals relative to neutral ones. Successful inhibitory control involves both reactive and proactive control mechanisms [5, 6, 7]: reactive inhibition involves stopping an action that is already in progress, while proactive inhibition involves preventing an action before it begins.

Here, to investigate reactive inhibition, we used the stop signal task (SST), which requires participants to initiate a response to a go stimulus and subsequently swiftly withhold their ongoing response when they encounter a stop signal [8]. The stop signal reaction time (SSRT) serves as an estimation of reactive inhibition performance [8], with lower SSRT corresponding to better action suppression. Participants performed an SST before and after the administration of one session of repetitive TMS (rTMS) over the preSMA/SMA, the rIFG, and the left (lM1). Results showed that rTMS over the preSMA/SMA improved the ability to inhibit prepotent action (i.e., better reactive inhibition) when emotional stimuli were presented. In contrast, reactive action suppression when neutral stimuli were presented was fostered by rTMS over the rIFG. No changes were observed after lM1 stimulation. These findings shed new light on the existence of partially separate neural pathways devoted to reactive inhibition in neutral or emotional contexts. However, the neural dynamics within the AIN and how these regions communicate to generate appropriate action inhibition are still unknown. Aron's original hypothesis proposed that the rIFG exerted influence on the right subthalamic nucleus (STN), with the preSMA being part of the same circuit [9].

In particular, during reactive inhibition, the rIFG and the preSMA engage the hyperdirect pathway of the basal ganglia (BG) [10]. The hyperdirect pathway consists of white matter tracts connecting the rIFG, the preSMA, and the STN and is involved in reactive response inhibition since it provides a rapid, global suppression of motor output [11, 12, 13, 14, 15, 16]. This causes inhibitory feedback over the motor cortices, which then cancels the initial go command [17, 18, 19, 20, 21]. Thus, it is reasonable to suppose that the connectivity between the rIFG, the preSMA/SMA, and M1 is crucial for action inhibition rather than activating a single node. However, previous studies used a correlational or a causal [3] approach that does not address the functional connectivity within the AIN. One way to define the interplay between these regions would be to assess which connections are causally involved in reactive inhibition and whether strengthening such connectivity (e.g., via exogenous brain manipulation) would lead to an enhancement in reactive inhibition. Our goal with the present work is to directly address this issue by employing a protocol called cortico–cortical paired associative stimulation (ccPAS; see [22, 23] for comprehensive reviews on the topic). This protocol consists of repeated paired stimulation of two interconnected brain areas with the aim of mimicking patterns of neuronal stimulation shown to induce spike‐timing–dependent plasticity (STDP)—a form of synaptic plasticity meeting the Hebbian principle that synapses are potentiated if the presynaptic neuron fires immediately before the postsynaptic neuron coherently and repeatedly [24, 25]. The ability of the ccPAS to potentiate premotor–motor connectivity has been demonstrated in several previous studies [26, 27, 28, 29, 30, 31, 32, 33, 34, 35, 36, 37, 38, 39, 40], which successfully demonstrated that it is possible to induce neurophysiological changes both online (i.e., during the ccPAS administration) and offline (i.e., after the ccPAS administration for ∼60 min), as well as behavioral improvements known to depend on the efficacy of the premotor–motor connectivity [29, 34, 39, 41]. Other ccPAS studies have successfully manipulated the connectivity between SMA and M1 [40, 42], demonstrating lasting (∼30 min) changes in corticospinal excitability (CSE) after the ccPAS administration. However, only a few ccPAS experiments investigated online changes in CSE [33, 34, 35, 38, 39, 40], or the influence of individual differences in motor excitability on the ccPAS effect [29]. Finally, only one ccPAS study aimed to ameliorate action inhibition through ccPAS administration within the AIN [43], resulting in a lack of improved performance in young individuals, an effect that was replicated by [44]. These studies boosted the connectivity between IFG and SMA, without taking into account their connectivity with M1, which is the area directly involved in suppressing the initial go command [17, 18, 19, 20, 21]. Here, we aimed to fill these gaps in the literature by boosting the connectivity between different nodes of the AIN with M1 (i.e., rIFG‐lM1, preSMA/SMA‐lM1, and rM1‐lM1) and by assessing online changes in CSE as well as behavioral improvements in a complex behavioral task such as the SST. Based on our previous TMS findings [4], which demonstrated the crucial role of the preSMA/SMA and rIFG in reactive inhibition, here we expected that enhancing their connectivity with M1 would result in a better inhibitory performance (i.e., shorter SSRT) in young healthy participants. Moreover, previous studies using the SST have shown mixed results regarding the influence of emotions on action inhibition—some reporting enhanced or diminished inhibition in response to emotional stimuli, while others found no significant effect [45]. To further investigate this issue, we employed negative and neutral stimuli as stop signals to examine whether emotionally salient cues preferentially engage distinct neural connections. Therefore, based on our prior findings [4], we hypothesized that the connection from the preSMA/SMA to M1 would be specifically involved in processing emotional stimuli. Moreover, in line with previous ccPAS studies that stimulated the SMA‐M1 connectivity [34, 40, 42], we expected an increment in MEPs amplitude following preSMA/SMA‐M1 stimulation.

## 2 Materials and Methods

### 2.1 Participants

A total of 52 healthy young adults were involved in the study. Participants (37 females and 15 males; 23.9 ± 2.3 years) were randomly assigned to one of three ccPAS conditions (preSMA/SMA‐lM1, rIFG‐lM1, rM1‐lM1). Specifically, 18 participants (12 females and six males; 24.7 ± 2.4 years) were randomly assigned to the preSMA/SMA‐lM1 group (one participant was then discarded because SST assumptions were violated, but her neurophysiological data were analyzed), 17 to the rIFG‐lM1 group (13 females and four males; 23.6 ± 2.0 years), and 17 to the rM1‐lM1 group (12 females and five males; 23.6 ± 2.3 years, see Table 1 for further demographic data). The number of participants was determined based on a power analysis, which indicated that a sample size of 15 participants is necessary to achieve a statistical power (1 − β) of 0.95 (two‐tailed *α* = 0.05; effect size *f* = 0.45 [46]; number of measurements = 4; correlation = 0.5). In each group of participants, MEPs were recorded during the ccPAS protocol in a subsample of 13 participants for each group (10 females and three males; 25.1 ± 2.7 years) for the preSMA/SMA‐lM1 group, (10 females and three males; 23.3 ± 2.1 years) for the rIFG‐lM1 group, and (10 females and three males; 24.2 ± 2.2 years) for the rM1‐lM1 group (See Table 2). Another power analysis was conducted, determining that a sample size of 12 participants is required to attain a statistical power (1 − β) of 0.99 (*α* = 0.05; effect size *f* = 0.86 [35, 47, 48]; number of measurements = 2; correlation = 0.5). All analyses were performed with G∗Power software [49]. Finally, groups were matched for age (*F*(2,49) = 1.52; *p* = 0.22; ηp^2^ = 0.06), and gender (*χ* ^2^ (2, *N* = 52) = 0.41; *p* = 0.81). All participants were right‐handed according to the Edinburgh Handedness Inventory [50], had normal or corrected‐to‐normal visual acuity in both eyes, and were naive as to the purposes of the experiment. None had neurological, psychiatric, or medical problems or any contraindication to TMS [51]. Participants provided written informed consent. The procedures were approved by the Bioethics Committee at the University of Bologna and were carried out in accordance with the ethical standards of the Declaration of Helsinki. No discomfort or adverse effects of TMS were reported or noticed during the experimental sessions. Furthermore, different personality states of the participants were investigated, as previous studies have shown that reactive inhibition may be influenced by personality traits commonly associated with psychological/psychiatric conditions (i.e., anxiety and impulsivity [45, 46, 52]). Subjective levels of anxiety were measured through the State‐Trait Anxiety Inventory (STAI; Trait‐Scale‐Y2) [53], and subjective levels of impulsivity were measured by the Barratt Impulsiveness Scale 11 (BIS‐11) [54]. The three groups did not show any significant difference in terms of anxiety (STAI‐Y2: *F*(2,49) = 0.54, *p* = 0.58, ηp^2^ = 0.02) and impulsivity scores (BIS‐11: *F*(2,49) = 0.22, *p* = 0.80, ηp^2^ < 0.01; see Table 1 for further details).

**TABLE 1:** Demographic data for each experimental group.

	preSMA/SMA‐lM1	rIFG‐lM1	rM1‐lM1
Age	24.7 ± 2.4	23.6 ± 2.0	23.6 ± 2.3
Gender	F = 12; M = 6	F = 13; M = 4	F = 12; M = 5
rMT	48.2 ± 9.3	46.2 ± 9.9	49.2 ± 7.2
STAI‐Y2	49.4 ± 5.6	47.3 ± 9.9	46.6 ± 6.6
BIS‐11	65.4 ± 5.7	64.3 ± 7	64.1 ± 6.2

*Note*: Age expressed as mean and standard deviation in years, resting motor threshold expressed as mean and standard deviation in % of maximum stimulator output. Gender is reported as the number of female and male participants. STAI‐Y2 and BIS‐11 are reported as mean standard deviation.

Abbreviations: BIS‐11, Barratt Impulsiveness Scale‐11; F, female; lM1, left motor cortex; M, male; preSMA/SMA, pre/supplementary motor cortex; rIFG, right inferior frontal gyrus; rM1, right motor cortex; rMT, resting motor threshold; STAI‐Y2, State‐Trait Anxiety Inventory.

**TABLE 2:** Demographic data of the MEP recording subsample.

	preSMA/SMA‐lM1	rIFG‐lM1	rM1‐lM1
Age	25.1 ± 2.7	23.3 ± 2.1	24.2 ± 2.2
Gender	F = 10; M = 3	F = 10; M = 3	F = 10; M = 3
rMT	46.8 ± 9.4	47.3 ± 10	47.7 ± 5.9
STAI‐Y2	49.4 ± 6	48.1 ± 10.9	48.4 ± 6.3
BIS‐11	64.4 ± 5.4	63.9 ± 7.8	63.6 ± 6.9

*Note*: Age expressed as mean and standard deviation in years, resting motor threshold expressed as mean and standard deviation in % of maximum stimulator output. Gender is reported as the number of female and male participants. STAI‐Y2 and BIS‐11 are reported as mean and standard deviation.

Abbreviations: BIS‐11, Barratt Impulsiveness Scale‐11; F, female; lM1, left motor cortex; M, male; preSMA/SMA, pre/supplementary motor cortex; rIFG, right inferior frontal gyrus; rM1, right motor cortex; rMT, resting motor threshold; STAI‐Y2, State‐Trait Anxiety Inventory.

### 2.2 Experimental Procedure

The experiments were programmed using MATLAB (version R2018b; TheMathWorks, Inc.) software to trigger TMS pulses, control stimulus presentation, and acquire behavioral responses. In each experiment, participants were randomly assigned to different groups according to the ccPAS protocol they would undergo. The main experiment involved three consecutive phases for each participant. Participants were seated in a quiet room ensuring that their position was centered relative to the screen and maintaining a viewing distance of 50 cm from the screen. At this point, the experimenter provided participants with an overview of the SST. This initial phase aimed to establish a baseline performance (i.e., pre‐ccPAS session), assessing the response inhibition ability of each participant. Subsequently, before the neurostimulation phase, the rMT was determined for each participant, followed by precise localization of the stimulation target on the scalp using a neuronavigation system with stereotaxic coordinates (see TMS paragraphs below for detailed procedures) and, finally, ccPAS was performed. Immediately afterward, in the final phase of the experiment (i.e., post‐ccPAS session), the same SST task was readministered to evaluate the effect of neurostimulation on response inhibition ability. Lastly, participants were asked to complete the personality traits questionnaires.

### 2.3 Stop Signal Task

**FIGURE 1:**
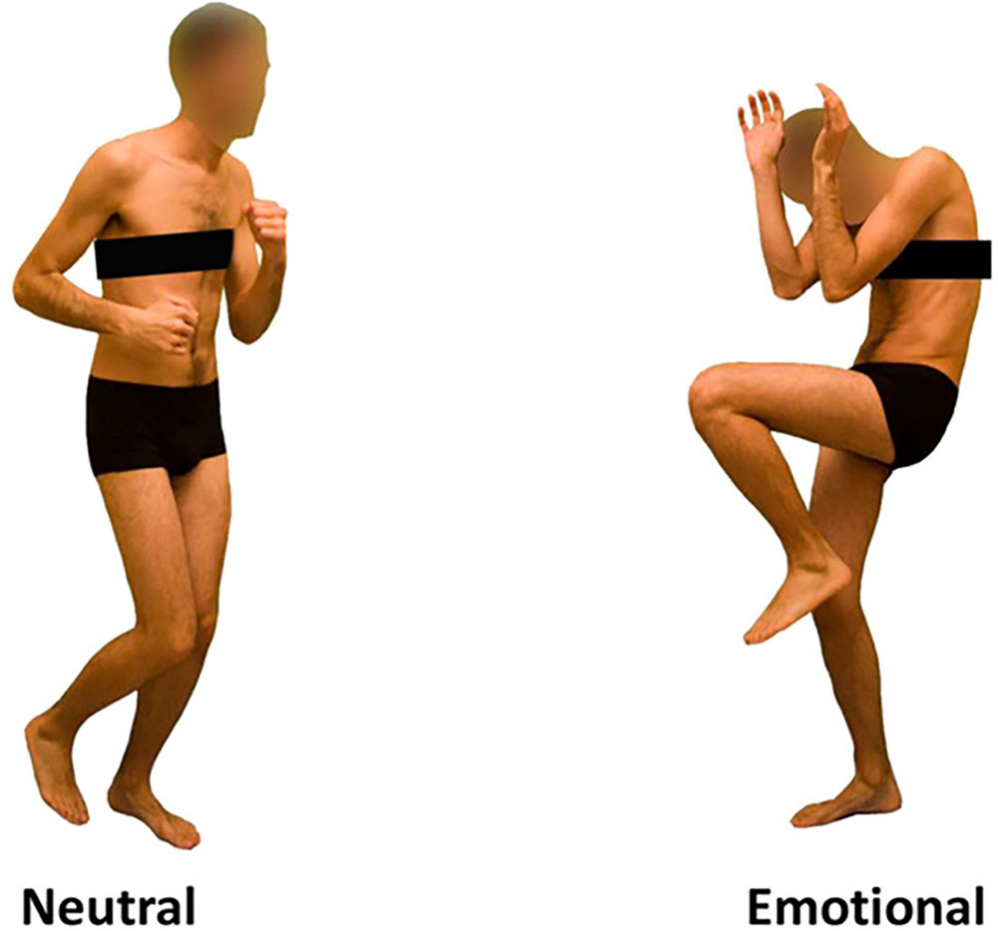
Visual stimuli used as stop signal stimuli. For all groups, stimuli consisted of two different body pictures with emotional and neutral body expressions that have been previously used and validated (see [58, 59]).

The SST comprises both go trials and stop trials [8, 55, 56, 57]. Go stimuli consisted of a black arrow pointing left or right, while stop stimuli could be one of two colored images of dynamic body postures [4, 58, 59]: a fearful emotional body posture acted as an emotional stop stimulus, while an image of a dynamic (i.e., running) neutral body posture was used as a control neutral stop stimulus (see Figure 1). The use of a neutral dynamic body stimulus matched for the conveyed implied motion is fundamental to prevent factors other than the emotional content from influencing our findings.

**FIGURE 2:**
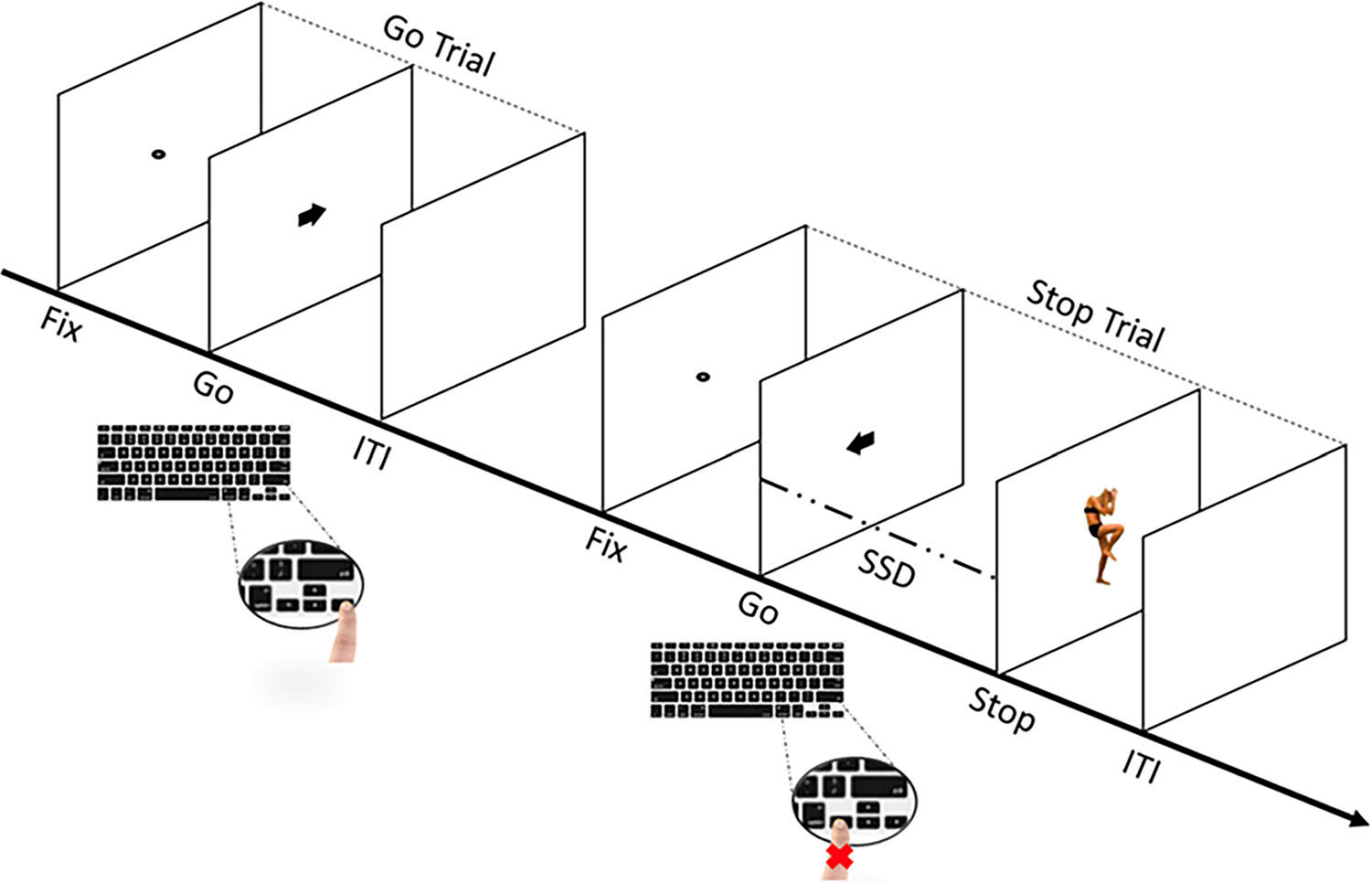
Sequence of trials in the stop signal task (SST). The experimental task includes both go and stop trials. In go trials, participants respond to the go stimulus (i.e., the direction of the arrow that appears on the screen) by pressing the corresponding arrow key on the keyboard. In stop trials, the arrow is followed by a stop signal after a variable stop signal delay (SSD) instructing participants to suppress the imminent go response. The initial value of the SSD was set to 250 ms and adjusted individually and dynamically throughout the experiment (i.e., staircase procedure), so that if participants successfully inhibited their response on a stop trial, the SSD was increased by 50 ms in a subsequent stop trial, while if they failed to withhold their motor response, the SSD was reduced by 50 ms in a subsequent stop trial. This image was adapted from [59]. Fix, fixation duration; ITI, intertrial interval.

Importantly, the two different body pictures (i.e., fearful and neutral expressions) were previously validated by Borgomaneri and colleagues in several studies [48, 60, 61, 62, 63, 64] or used by other research groups [65, 66, 67, 68, 69, 70] and, importantly, have also been used as stop signals in other studies [4, 58, 59]. Participants started the SST by performing a short practice block (approximately 3 min, 32 trials) to familiarize themselves with the task. Immediately afterward, they performed four experimental blocks that constituted the main task. Each block was composed of a total of 128 trials, consisting of 96 go trials (75%) and 32 stop trials (25%). Therefore, during the whole task, each participant was presented with a total of 384 go trials and 128 stop trials. Each trial began with a black dot centered on a blank white screen for 400 ms (serving as a fixation point) and ended with an empty white screen for a randomly determined interval between 1000 and 2000 ms, which acted as the intertrial interval. During go trials, participants were required to press the left key as quickly as possible when a black arrow pointing to the left appeared, and the right key when the arrow pointed to the right, using their right hand. If no response was made, the stimulus remained on the screen for a maximum of 750 ms. Stop‐signal trials started as go trials but included an additional stop signal, which was a picture stimulus presented for 100 ms after a variable stop signal delay (SSD) from the onset of the go stimulus (i.e., the arrow). This signal instructed participants to suppress their imminent response. The initial SSD value was set to 250 ms and was adjusted individually and dynamically throughout the experiment, using a staircase procedure. If participants successfully inhibited their response, the SSD increased by 50 ms for the next stop trial; if they failed, the SSD decreased by 50 ms [46, 52, 57, 71, 72]. In any case, the SSD remained within a range from a minimum of 50 ms to a maximum of 750 ms. This staircase adjustment ensured approximately 50% successful inhibition, calculated independently for each stop signal and participant [57, 73, 74]. Participants were instructed to respond as quickly and accurately as possible to the arrows and to attempt to inhibit their response when the stop signal appeared after the initial go stimulus. They were informed that it might not always be possible to inhibit their response and that they should continue the task even if they made an error [46, 57]. Additionally, participants were advised not to hesitate or slow down to increase their chances of stopping. The task design followed the recommendations of Verbruggen and colleagues [57] (see Figure 2).

### 2.4 ccPAS Protocol

TMS was administered using two 50‐mm butterfly shaped iron‐branding coils. In all groups, we administered 90 pairs of TMS pulses at a rate of 0.1 Hz for 15 min [22]. Each participant's rMT was assessed using the established procedure as the minimum stimulator output intensity able to induce MEPs > 0.05 mV in five out of 10 consecutive trials. In all participants, rMT was assessed immediately before the ccPAS protocol. A one‐way ANOVA on rMT on the entire sample revealed no significant effects of group (*F*(2,49) = 0.51; *p* = 0.60; ηp^2^ = 0.02; preSMA/SMA‐lM1: mean ± SD 48.78 ± 9.90; rIFG‐lM1: 46.23 ± 9.85; rM1‐lM1: 49.23 ± 7.16; see Table 1). For the subsample utilized for the MEP analysis, another one‐way ANOVA on rMT intensity showed no significant effect of group (*F*(2,36) = 0.03; *p* = 0.96; ηp^2^ < 0.01; preSMA/SMA‐lM1: 46.84 ± 9.39; rIFG‐lM1: 47.30 ± 9.99; rM1‐lM1: 47.69 ± 5.86; see Table 1). In the preSMA/SMA‐lM1 group, a first pulse was administered over the preSMA/SMA and the second pulse was administered over the left M1 with an interstimulation interval (ISI) of 6 ms, so to activate short‐latency preSMA/SMA‐to‐lM1 connections [42, 75, 76, 77, 78]. In the rIFG‐lM1 group, the first pulse was delivered over the rIFG 6 ms before the second pulse, which was administered over the lM1 [78, 79]. Finally, in the rM1‐lM1, the first pulse was delivered over the rM1 8 ms before the second pulse, which was delivered over the left M1, which is the optimal ISI to induce interhemispheric inhibition [80, 81, 82]. In preSMA/SMA‐lM1 and rIFG‐lM1 groups, the first pulse intensity was set to 90% of the individual's rMT, while the M1 stimulation was adjusted to evoke ∼1 mV MEPs, in line with several ccPAS studies that have demonstrated the effectiveness of subthreshold conditioning [29, 83, 84]. In the rM1‐lM1 group, both pluses were delivered with a suprathreshold intensity [80]. Importantly, all M1 stimulations during the ccPAS were performed using suprathreshold TMS intensity, specifically with an intensity able to evoke ∼1 mV MEPs, so that on each paired‐stimulation we induced a MEP recorded through Ag/AgCl surface electrodes placed in a belly‐tendon montage over the relaxed right first dorsal interosseous (FDI), allowing to track the emergence of a gradual enhancement of corticomotor excitability during ccPAS. Electromyography (EMG) signals were acquired using a Biopac MP‐35 (Biopac, USA) electromyograph, band‐pass filtered between 30 and 500 Hz and sampled at 10 kHz. TMS pulses were remotely administered using a MATLAB script (version R2022b; TheMathWorks, Inc.).

### 2.5 Neuronavigation

**FIGURE 3:**
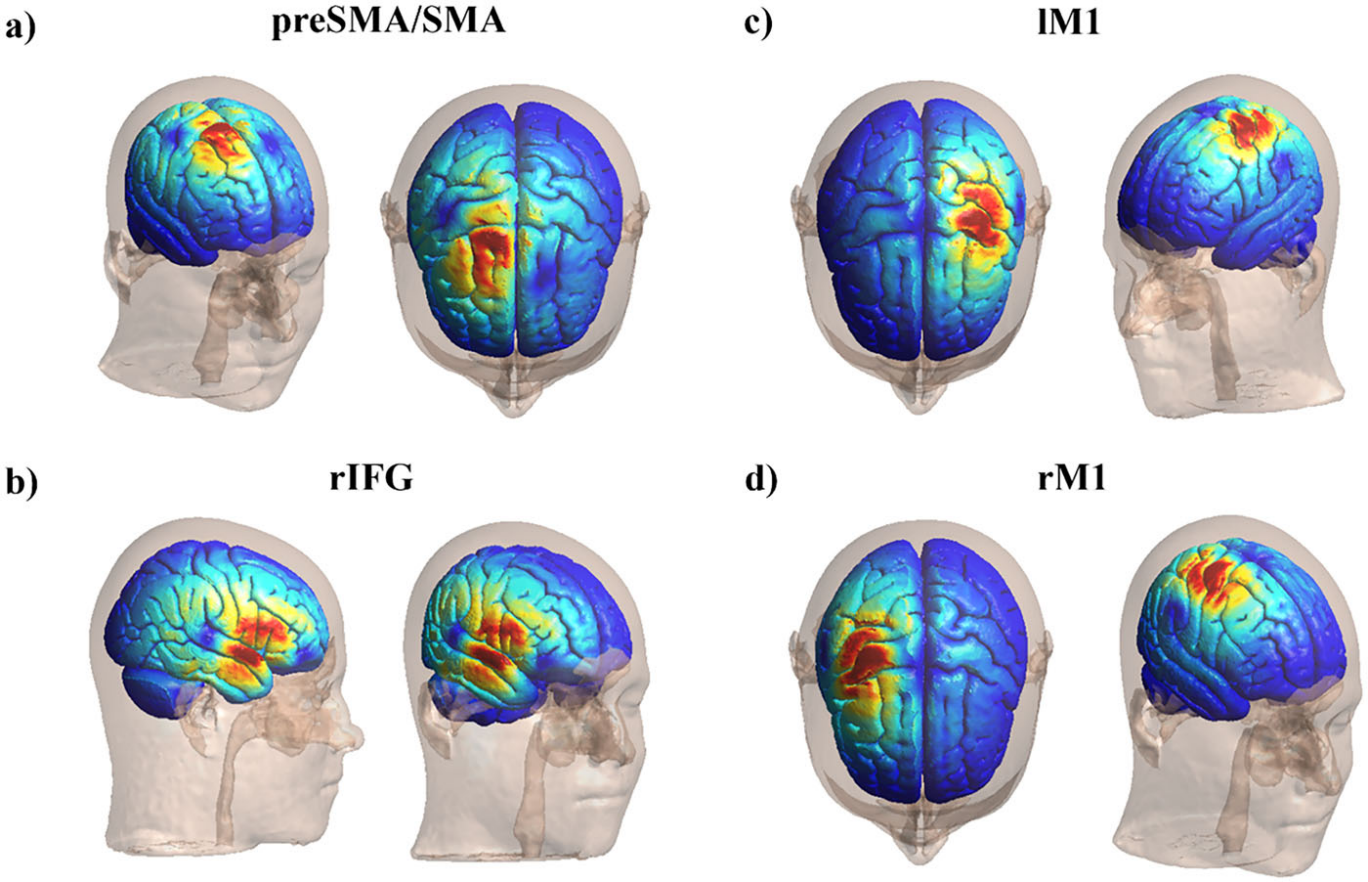
Computational simulations of the estimated electric field distribution transcranial magnetic stimulation (TMS) targeting the brain. The volumetric spread of magnetic field simulation was created using SimNIBS v4.0.1. Conductivities for different tissue compartments were set as follows: 0.465 S/m (skin), 0.01 S/m (skull), 0.5 S/m (eyeballs), 1.654 S/m (cerebrospinal fluid), 0.275 S/m (gray matter), and 0.126 S/m (white matter). The coils were placed according to the mean coordinates of each area. The estimated computational simulation showed an estimated propagation of the TMS stimulation over the chosen sites, hence supporting the TMS setup used. preSMA/SMA, presupplementary/supplementary motor area; rIFG, right inferior frontal gyrus; r/lM1, right or left primary motor cortex.

Brain target areas were identified using the EMS SofTaxic Navigator system [85, 86], which estimates Talairach coordinates from a stereotaxic template constructed via magnetic resonance imaging (MRI). Skull landmarks and approximately 80 points providing a uniform scalp representation were digitized using a Northern Digital Polaris Vicra digitizer [4, 87, 88, 89, 90]. Each participant's individual MRI was estimated through a 3D warping procedure that fits a high‐resolution MRI template to the participant's scalp model and craniometric points, ensuring a global localization accuracy of around 5 mm [87]. The preSMA/SMA and the rIFG were targeted using the following Talairach coordinates: *x* = 9, *y* = 6, *z* = 49 for preSMA/SMA [91, 92] and *x* = 51, *y* = 15, *z* = 1 for rIFG [91]. The lM1 and rM1 were defined as the location where stimulation consistently elicited the largest motor‐evoked potentials (MEPs) in the contralateral FDI muscle [51]. The SofTaxic Navigator system was used to verify the projection of the targeted scalp positions onto the brain surface, confirming correct coil placement at all sites (see Figure 3 for the computational estimation of the TMS pulse propagation on the stimulation sites). The Talairach coordinates for the lM1 and rM1 (the optimal scalp position for both right and left FDI) were as follows (mean ± SD): *x* = −31.8 ± 8.9, *y* = −20.1 ± 20.7, *z* = 55.4 ± 9.9 for lM1 and *x* = 35.4 ± 8.5, *y* = −20.8 ± 18.2, *z* = 52.2 ± 7.4 for rM1. For the preSMA/SMA, the coordinates were as follows: *x* = 13.1 ± 12.1, *y* = 9.8 ± 7.7, *z* = 56.6 ± 9.0. For the rIFG, the coordinates were as follows: *x* = 47.3 ± 8.3, *y* = 10.9 ± 6.5, *z* = 3.5 ± 6.6.

### 2.6 Behavioral and Neurophysiological Data Processing

To assess participants' performance on the SST, the SSRT, an index of reactive inhibition, was estimated following Logan's race‐model concept [8]. SSRT is the overall latency of a chain of processes involved in stopping a response, including the detection of the stop signal. However, prior to analyzing SSRT, the reliability of the overall performance of the participants in the task was verified by calculating the inhibition rate, which must be around 50% [93]. Furthermore, we made sure that reaction times (RTs) for unsuccessful stop trials were shorter than go trial RTs [57]. Subsequently, data collected in this experiment were processed to estimate SSRTs [93]. Accordingly, data were analyzed by adopting the integration method with the replacement of go omissions. In particular, the point at which the stop process ends is estimated by integrating the RT distribution and finding the point at which the integral is equal to *p*(respond|signal). The ending time of the stop process corresponds to the *n*th RT, where *n* is the number of RTs in the RT distribution of go trials multiplied by *p*(respond|signal). Also, to determine the *n*th RT, all go trials with a response were considered, including go trials with a choice error and go trials with a premature response. It is important to highlight that these omissions (i.e., go trials in which participants did not respond before the end of the trial) were assigned the maximum RT to compensate for the lack of response. Moreover, premature responses in unsuccessful stop trials (i.e., responses executed before the stop signal is presented) were included in calculating *p*(respond|signal) and mean SSD. This version of the integration method yields the most reliable and unbiased SSRT estimation (for further details and an extensive review, see [57]). Furthermore, we verified that the assumptions of the independent race model were respected [74, 93]. SST data collected in both sessions do not violate any assumption of the independent race model (see Table 3 for descriptive SST data). Thus, it was possible to reliably estimate the SSRT values.

**TABLE 3:** Descriptive information of the stop signal task (SST).

pre‐ccPAS	preSMA/SMA‐lM1		rIFG‐lM1		rM1‐lM1	
	Fearful	Neutral	Fearful	Neutral	Fearful	Neutral
Inhibition rate (%)	47.2 ± 4.9	47.2 ± 3.8	48.9 ± 2.8	48.4 ± 3.7	48.3 ± 4.1	48.3 ± 3.6
SSD (ms)	164 ± 64.1	175 ± 62.9	212 ± 94	207 ± 96.6	225 ± 76.7	223 ± 79.3
SSRT (ms)	235 ± 32.5	224 ± 26.6	200 ± 25.2	206 ± 32.8	217 ± 30.6	218 ± 34.4
Unsucc RT (ms)	361 ± 65.5	360 ± 61.4	374 ± 65	374 ± 69.4	392 ± 51.7	398 ± 60
Go correct (%)	94.9 ± 6.3		95.5 ± 3.8		95.8 ± 3.8	
**post‐ccPAS**	**preSMA/SMA‐lM1**		**rIFG‐lM1**		**rM1‐lM1**	
	Fearful	Neutral	Fearful	Neutral	Fearful	Neutral
Inhibition rate (%)	48.3 ± 2.4	48.3 ± 2.9	48.4 ± 3.3	48.5 ± 2.8	48.3 ± 2.2	48.2 ± 2.4
SSD (ms)	178 ± 68.2	185 ± 73.9	204 ± 81.7	204 ± 83	204 ± 64.6	208 ± 64.4
SSRT (ms)	223 ± 28.3	216 ± 27.4	209 ± 25.7	208 ± 22.2	226 ± 25.6	222 ± 26.6
Unsucc RT (ms)	364 ± 65.5	371 ± 72.2	374 ± 68.6	372 ± 65.4	387 ± 48.3	386 ± 54.1
Go correct (%)	96.5 ± 4.5		96.8 ± 1.7		96.7 ± 4	

*Note*: For each group and session, inhibition rate, stop signal delay (SSD), stop signal reaction time (SSRT), unsuccessful stop reaction time (Unsucc RT), and go correct are depicted in the table.

Abbreviations: ccPAS, cortico–cortical paired associative stimulation; lM1, left motor cortex; preSMA/SMA, pre/supplementary motor cortex; rIFG, right inferior frontal gyrus; rM1, right motor cortex.

To characterize changes in inhibitory control resulting from neurostimulation for each specific network, we created an inhibitory performance index of SSRT (ΔSSRT). Specifically, ΔSSRT was calculated for each participant by considering SSRT collected after neurostimulation minus the SSRT collected before neurostimulation (i.e., post‐ccPAS − pre‐ccPAS, mean of the emotional and neutral stop signals). This approach allows us to have a direct assessment of changes in inhibitory capacity associated with the precise cortical network targeted by the ccPAS. MEPs were assessed by measuring peak‐to‐peak EMG amplitude (in mV). Then, we analyzed peak‐to‐peak MEP amplitudes induced by lM1 stimulation in the FDI muscle during the ccPAS session automatically extracted from EMG signals using a custom MATLAB script (MathWorks, USA) and measured in mV. Trials showing EMG activity 100 ms prior to TMS were discarded from further analysis (4.4%) as well as MEPs due to environmental noise interference (0.2%). Additionally, MEPs with a peak‐to‐peak amplitude less than 0.05 mV were also excluded (4.4%). Lastly, a MEP modulation index was computed as the difference between the last and the first 10 MEPs [39]. Data were analyzed offline using custom‐made MATLAB scripts (version R2022b; TheMathWorks, Inc.).

### 2.7 Statistical Analysis

Mixed‐design ANOVAs were used to investigate differences within and between groups. All the post hoc analyses were conducted with the Duncan test, and the significance threshold was set at *p* < 0.05.

Additionally, to investigate whether neurophysiological indices of Hebbian plasticity were correlated to the magnitudes of behavioral changes following ccPAS in the preSMA/SMA‐lM1 group, we computed a Pearson correlation coefficient with the MEP modulation index and the ΔSSRT. Finally, to test whether interindividual differences in the behavioral effect of ccPAS (i.e., reduction in the SSRT in the post relative to the pre‐ccPAS session) correlated with global indices of motor excitability (i.e., the rMT), an additional Pearson correlation coefficient was computed. Finally, a multiple linear regression was conducted to examine whether interindividual differences in the behavioral effect of ccPAS can be ascribed to interindividual differences in personality traits. All statistical analyses were performed with STATISTICA (StatSoft STATISTICA 13).

## 3 Results

### 3.1 Verification of the SST Assumptions

We assessed the differences between the mean RT on unsuccessful stop trials (i.e., trials in which the participants failed to inhibit their responses) for both stop trials with the emotional stimulus and the neutral one as stop signals and the mean RT registered during go trials. We performed a 3 × 2 × 3 ANOVA on RT with Trial type (go RT/unsuccessful emotional/unsuccessful neutral) and Session (pre‐ccPAS/post‐ccPAS) as within‐subject factors and Group (preSMA/SMA‐lM1/rIFG‐lM1/rM1‐lM1) as between‐subject factor. The results showed a significant effect of Trial type (*F*(2,96) = 0.70, *p* < 0.01, ηp^2^ = 0.82). Post hoc analysis revealed that RT in go trials were significantly longer than both unsuccessful emotional (*F*(2,48) = 2.54, *p* < 0.01, *d* = 0.66) and unsuccessful neutral RT during stop trials (*F*(2,48) = 2.54, *p* < 0.01, *d* = 0.64). Then, we verified the successful implementation of the staircase procedure, responsible for maintaining the inhibition rate (i.e., percentage of successful inhibitions during stop trails) at an approximate value of 50%. We conducted a 2 × 2 × 3 ANOVA on the percentage of successful stop trials with Stop type (emotional/neutral) and Session (pre‐ccPAS/post‐ccPAS) as within‐subject factors and Group (preSMA/SMA‐lM1/rIFG‐lM1/rM1‐lM1) as a between‐subject factor. The analysis revealed no significant differences between Groups (*F*(2,48) = 0.36, *p* = 0.69, ηp^2^ = 0.01), Session (*F*(2,48) = 0.46, *p* = 0.50, ηp^2^ = 0.01), and Stop type (*F*(2,48) = 0.20, *p* = 0.66, ηp^2^ < 0.01). Furthermore, no interactions were found significant (all *F*(2,48) ≤ 1.17, all *p* ≥ 0.32, all ηp^2^ ≤ 0.04). Lastly, we conducted a 2 × 3 ANOVA on the percentage of correct responses during go trials with Sessions (pre‐ccPAS/post‐ccPAS) as a within‐subject factor and Group (preSMA/SMA‐lM1/rIFG‐lM1/rM1‐lM1) as a between‐subject factor. A significant main effect of Session was found (*F*(2,48) = 9.55, *p* < 0.01, ηp^2^ < 0.01). Post hoc analysis revealed that the percentage of correct go trials increased in the post‐ccPAS session (*F*(1,49) = 1.17, *p* < 0.01, *d* = 0.30), due to a learning effect. In conclusion, SST data collected in both sessions do not violate any assumption of the independent race model (see Table 3 for descriptive SST data). Thus, it is possible to reliably estimate the SSRT values [57].

### 3.2 Improved SSRT Performance in the PreSMA/SMA‐lM1 Group

**FIGURE 4:**
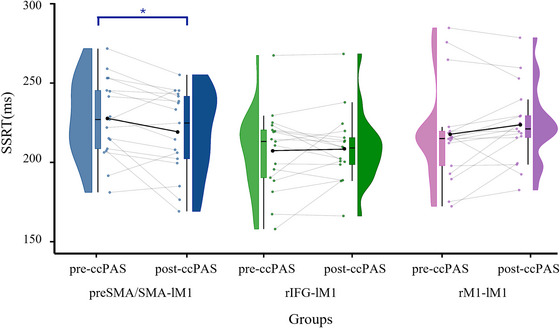
Graph representing the Group × Session interaction. The half‐violin plots and associated box plots represent the visual distribution of the data pre‐ and post‐ccPAS. In light and dark blue, the preSMA/SMA‐lM1 group showed a significant improvement in behavioral inhibition (i.e., shorter SSRT), while the rIFG‐lM1 and rM1‐lM1 groups, represented by light and dark green and light and dark violet, respectively, did not. The black dots represent the mean value of each distribution, while the whiskers extend to the most extreme values within 1.5 times the interquartile range (IQR) beyond the quartiles. Asterisks indicate significant comparisons (*p* < 0.05). ccPAS, cortico‐cortical paired associative stimulation; preSMA/SMA, presupplementary/supplementary motor area; rIFG, right inferior frontal gyrus; r/lM1, right or left primary motor cortex; SSRT, stop signal reaction time.

To verify the main hypothesis of the present study, SSRT data were analyzed using a 2 × 2 × 3 ANOVA with Stop type (emotional/neutral) and Session (pre‐ccPAS/post‐ccPAS) as within‐subject factors, and Group (preSMA/SMA‐lM1/rIFG‐lM1/rM1‐lM1) as between‐subject factor. The analysis revealed a significant Group × Stop type interaction (*F*(2,48) = 4.29, *p* = 0.02, ηp^2^ = 0.15). More interestingly, a significant Group × Session interaction was found (*F*(2,48) = 3.98, *p* = 0.03, ηp^2^ = 0.14). The post hoc analysis showed a significant difference between the pre (229.29 ± 29.81) and the post‐ccPAS session (219.49 ± 27.67) selectively in the preSMA/SMA‐lM1 group (*p* = 0.05, *d* = 0.34), while the same contrast was not found to be significant in either of the other groups (all *p* > 0.21; see Figure 4). No other main effects or interactions resulted significant (all *F* < 2.63; all *p* > 0.08). To further investigate the effect of neurostimulation on the SSRT data mean of the two stop signals, we compared ΔSSRT with 0 (indicating no variation between Sessions, pre‐ccPAS vs. post‐ccPAS) using one‐sample *t*‐tests. These tests revealed that the preSMA/SMA‐lM1 group showed significantly reduced SSRT (*t*(16) = −2.64, *p* = 0.01, *d* = 0.64). On the contrary, the same analysis revealed no changes in the SSRT in the other two groups (all *p* > 0.14).

### 3.3 Increment in MEPs Amplitude in the preSMA/SMA and rM1‐lM1 Groups

**FIGURE 5:**
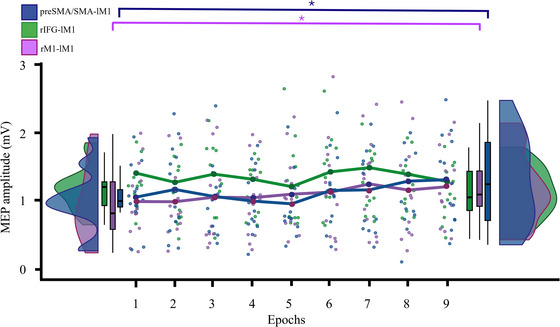
Time course of motor evoked potential (MEP) amplitudes collected during the ccPAS protocol. MEPs were divided into nine epochs, each consisting of 10 MEPs. Dots represent the distribution of MEP amplitudes for each epoch, color‐coded by group, while lines show the time course of average MEP amplitudes across epochs for each group. Each point on the lines corresponds to the average amplitude of a given epoch. On the sides, half‐violin and box plots illustrate the MEP distributions of the first and last epochs. The horizontal line in the boxplot represents the median of the distribution, while the whiskers extend to the most extreme values within 1.5 times the interquartile range (IQR) beyond the quartiles. The graph shows a significant increase in MEP amplitude during the last epoch for the rM1‐lM1 and preSMA/SMA‐lM1 groups compared with the first epoch, with asterisks marking significant differences (*p* < 0.05). preSMA/SMA, presupplementary/supplementary motor area; rIFG, right inferior frontal gyrus; r/lM1, right or left primary motor cortex.

MEPs recorded during the ccPAS protocol were clustered into nine bins, each containing 10 MEPs (0–90). This division was made to capture the temporal dynamics of MEP changes throughout the experiment, allowing us to observe potential trends across different phases. The progression of MEPs across these bins was analyzed separately for each stimulation group: preSMA/SMA‐lM1, rM1‐lM1, and rIFG‐lM1. As can be seen in Figure 5, in the preSMA/SMA‐lM1 and rM1‐lM1 groups, smoothed MEPs distribution showed a gradual enhancement that accurately fit a linear distribution [preSMA/SMA‐lM1: f(*x*) = 0.0026 × *x* + 1.0466; *R* ^2^ = 0.41; rM1‐lM1: f(*x*) = 0.0027 × *x* + 0.9996; *R* ^2^ = 0.90], whereas no consistent change was observed in the rIFG‐lM1 group [f(*x*) = −0.0004 × *x* + 1.3668; *R* ^2^ = 0.02]. A Bin (0–10 and 81–90) × Group ANOVA showed a marginally significant main effect of the factor Bin (*F*(1,36) = 3.84, *p* = 0.06, ηp^2^ = 0.10). More interestingly, a Bin × Group interaction was found (*F*(2,36) = 4.06, *p* = 0.03, ηp^2^ = 0.18). Post hoc analysis showed higher MEPs in the last 10 MEPs compared to the first 10 MEPs in both the preSMA/SMA‐lM1 and the rM1‐lM1 groups (all *p* ≤ 0.05), whereas no difference between MEPs at the end and the beginning of the protocol was observed in the rIFG‐lM1 group (*p* = 0.27; Figure 5).

### 3.4 Variability in the Behavioral Effect of ccPAS and Its Relation to Motor Excitability

**FIGURE 6:**
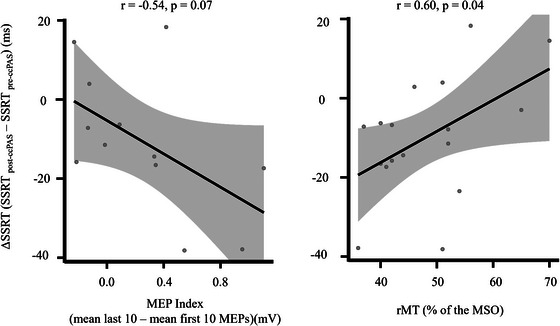
Correlation between physiological indices and stop signal reaction time (SSRT). Correlation plot between the ΔSSRT (on the *y*‐axis) and individual physiological indices (on the *x*‐axis) in the preSMA/SMA‐lM1 group. ΔSSRT is calculated by subtracting the mean SSRT in the pre‐ccPAS session from the mean SSRT in the post‐ccPAS session. On the left, the correlation between ΔSSRT and MEP index (*r* = −0.54, *p* = 0.07), calculated by subtracting the mean of the first 10 MEPs from the mean of the last 10 MEPs recorded during the ccPAS. On the right, the correlation between ΔSSRT and rMT (*r* = 0.60, *p* = 0.04), assessed before the stimulation. On top of both graphs, Pearson's *r* and *p* values are indicated. ccPAS, cortico‐cortical paired associative stimulation; MEP, motor evoked potential; rMT, resting motor threshold; MSO, maximum stimulator output; SSRT, stop signal reaction time.

To test for a relation between the neurophysiological and behavioral data, we computed a Pearson correlation between the MEP index and the ΔSSRT. We found that the MEP index was marginally significantly correlated with the behavioral effect found in the preSMA/SMA‐lM1 group (*r* = −0.54, *p* = 0.07; Figure 6), with MEPs increase associated with better reactive inhibition following the preSMA/SMA‐lM1 ccPAS protocol. The same analysis conducted on the rM1‐lM1 group (which also showed MEPs increase following the ccPAS administration) was not significant (*r* = −0.06, *p* = 0.86). These findings suggest the existence of a link between Hebbian plasticity we have induced stimulating the preSMA/SMA and M1 connectivity and behavioral advantages in action suppression. Building on previous studies [29], we also tested whether individual's rMT predicted behavioral differences after the preSMA/SMA‐lM1 ccPAS. We found that rMT significantly predicted the ΔSSRT (*r* = 0.60; *p* = 0.04; Figure 6), indicating that individuals in the preSMA/SMA‐lM1 group with more excitable motor systems tended to show greater SSRT improvements following preSMA/SMA‐lM1 ccPAS administration.

### 3.5 Absence of a Relationship Between Changes in Reactive Inhibition and Personality Traits

A multiple linear regression analysis was conducted to assess whether STAI‐Y2 and BIS‐11 scores significantly predicted the improvement in inhibitory control in the preSMA/SMA‐lM1 group (i.e., ΔSSRT). The overall model was not statistically significant, accounting for only 2.3% of the variance in the outcome variable (*R* ^2^ = 0.02; adjusted *R* ^2^ = −0.18). None of the predictors significantly contributed to the model (all *p* > 0.61), a result in line with previous literature suggesting that the correlation between questionnaire‐based impulsivity and performance‐based measures tends to be poor or null [94, 95].

## 4 Discussion

Halting inappropriate actions is pivotal to producing adequate behavioral responses. Despite the importance of such complex cognitive function, the neural dynamics subtending such ability are still unknown. Although previous studies have successfully demonstrated the crucial role of preSMA/SMA and the rIFG in reactive inhibition (see [45] for a review on the topic), how these regions interact with M1 to ultimately interrupt an ongoing action and whether reactive inhibition can be ameliorated by boosting the AIN connectivity is still obscure. To address this question, using the ccPAS protocol, we targeted the connections between preSMA/SMA and M1 and between the rIFG and M1 to investigate whether such connections are functionally malleable and crucial for action suppression. We expected the increment in connectivity through the ccPAS to induce an increased MEP amplitude as well as a behavioral improvement in reactive inhibition, measured using the SST after the ccPAS stimulation. Our results revealed that the ccPAS protocol can induce STDP in both the rM1‐lM1 as well as the pre‐SMA/SMA‐lM1 groups, suggesting a progressive enhancement of excitatory (rather than inhibitory) preSMA/SMA‐to‐M1 and rM1‐to‐lM1 interactions, due to the gradually increasing efficacy of excitatory synaptic input to lM1 neurons. The increased CSE after ccPAS between the two motor cortices may be explained by the reduction of the interhemispheric inhibitory (IHI) mechanisms caused by the long‐term potentiation (LTP) effects of the stimulation [80, 81, 82, 96], while the increased CSE after the preSMA/SMA‐to‐M1 ccPAS is in line with previous paired‐pulse TMS studies investigating such functional connectivity both at rest [89, 97, 98, 99] and during action reprogramming [75], demonstrating the existence of a preSMA/SMA to M1 excitatory connectivity as well as with ccPAS studies demonstrating its functional malleability [40, 42]. Such findings follow results obtained in nonhuman primates, in which electrical stimulation delivered to the SMA evokes excitatory responses in M1 [100] and with pharmacological studies indicating that the excitatory response of M1 following SMA electrical stimulation is mediated by glutamatergic interactions [101, 102, 103]. Interestingly, only the preSMA/SMA‐to‐M1 connectivity appears to be specifically involved in reactive inhibition, as behavioral improvements in task performance (i.e., reduced SSRT) have been observed selectively following the potentiation of this pathway. Furthermore, we found a marginally significant relationship between behavioral and physiological changes: greater ccPAS‐induced MEP facilitation predicted enhanced action control following preSMA/SMA‐to‐M1 ccPAS. Therefore, this finding aligns with previous studies demonstrating a link between physiological modulation and behavioral outcomes [29, 38, 40]. Overall, our findings highlight the causal role of the preSMA/SMA to M1 connectivity in mediating action inhibition. It is important to mention that although a basal ganglia/subthalamic route might mediate important aspects of action inhibition [104], even the most direct path through this route is associated with neuronal latencies above the 6 ms preSMA/SMA/M1 latency [105]. PreSMA/SMA was already demonstrated to be important in situations involving direct competition [106], updating [107], reprogramming [108], and stopping actions reactively (for a systematic review see [3]) as well as proactively [109, 110, 111, 112, 113, 114]. Indeed, the specific involvement of the preSMA in an SST has been demonstrated by [115], in which authors used the SSRT as an index of action suppression, and they found that higher activation in the preSMA was associated with shorter SSRTs (i.e., more efficient performance). In addition, human patient studies revealed that damage to the preSMA/SMA region elongated subjects' SSRT [116] (for a review see [117]). All these findings corroborated the role of preSMA/SMA in action inhibition without clarifying its influence on M1. Previous work has shown that rTMS over preSMA/SMA during response conflict results in greater activation in M1 controlling the competing response [118], and a recent study has shown latency‐ and volume‐dependent influences of SMA onto M1 [99], indicating that the SMA can influence M1. Indeed, preSMA/SMA activity was found to correlate with CSE reductions suggesting top‐down influences of preSMA over response channels during proactive action stopping [119]. Thus, here we replicated previous findings indicating the existence of functionally malleable and excitatory connections between preSMA/SMA and M1, and we additionally demonstrated that these connections are crucial for reactive inhibition. Interestingly, a recent ALE meta‐analysis [2] focusing on the brain activations related to go/nogo and SST revealed a convergence cluster for standard SST and go/nogo tasks, which lay anterior to the posterior preSMA/SMA region, providing further evidence for a functional gradient along the rostro‐caudal axis, with more anterior regions being involved in cognitively more demanding control processes for selecting the correct response alternative relative to the more posterior portions, suggesting the crucial role of our selected region in the SST.

Our results support previous studies that successfully reported behavioral improvement after ccPAS both stimulating the motor/premotor areas as well as the visual system (for a recent review, see [22]). However, here we demonstrated the possibility of ameliorating a complex executive function such as action inhibition, which represents a crucial cognitive function, widely impacted in clinical and psychiatric populations (see [45] for a comprehensive review on the topic). Based on our previous findings [4] and in line with a paired‐pulse TMS study [120], which demonstrated increased CSE stimulating the preSMA/SMA‐M1 connectivity, selectively observing negative stimuli, we expected an emotion‐specific effect (i.e., reduced SSRT selectively for fearful‐related stop signals). However, our findings demonstrated a general increase in action inhibition unrelated to the presented stop stimulus.

However, it is worth noting that inhibiting an area via rTMS is largely different from increasing the connectivity of the same area with another. The increased connectivity between preSMA/SMA and M1 may have increased the descendent inputs on the STN. Structural and functional neuroimaging studies, as well as deep brain stimulation and neuropharmacological studies, pointed out STN as a fundamental area involved in integrating cognitive, motor, and emotional components of complex behavioral responses [121, 122, 123, 124, 125, 126, 127, 128]. Thus, preSMA/SMA‐M1 incremented connectivity may result in a more efficient top‐down error monitoring [123, 129, 130, 131], reducing the effect of emotional components of the stimulus over STN and the hyperdirect pathway. To further investigate this lack of an emotion‐specific effect, future studies should investigate whether changing the role of the emotional stimulus (e.g., presented as stop signal, go signal, or presented before the go signal as a prime), but also the relevance of the emotional stimulus (i.e., task‐relevant, see [132, 133, 134] or task‐irrelevant, see [46, 72, 135]), would induce any emotion‐specific effect after the ccPAS stimulation. Moreover, future studies should employ a positive stimulus or an additional negative control stimulus (e.g., an angry body posture) to assess potential effects related to valence or arousal. However, previous findings [58, 59] indicate no significant difference between positive and negative stimuli in modulating the SSRT when used as stop signals.

Finally, as expected, individual differences in the global measure of motor excitability (i.e., rMT) predict sensitivity to associative plasticity [29]. Our findings show that individuals with more excitable motor systems display better behavioral performances [136, 137] as well as larger behavioral improvements following ccPAS, confirming and expanding previous work showing that inter‐individual differences in rMT predict the magnitude of LTP effects in the motor system [138] and TMS‐induced behavioral effects [29]. Interestingly, the same ISI between rIFG and M1 did not produce any behavioral improvement or effect on MEP amplitude. The lack of behavioral effects is in line with several studies that have proposed that the rIFG is not exclusively involved in inhibitory control, but rather forms part of a broader frontoparietal network that supports general cognitive control processes [139, 140, 141]. Furthermore, a recent study found that excitatory rTMS applied to the rIFG did not influence reactive inhibition [142], which may suggest the need for a revision of the original hypothesis proposed by Aron and colleagues [9, 143, 144]. Indeed, our absence of effect may be explained by the functional interplay between preSMA/SMA, rIFG, and M1. According to a previous study [145], preSMA activity precedes rIFG during response inhibition and mediates rIFG‐to‐M1 connections, suggesting the preSMA area covers a gating function over M1 [146, 147]. Notably, a study using diffusion MRI tractography suggested that rIFG may be responsible for increasing the excitatory influence of preSMA/SMA over the STN [148], resulting in an increment in behavioral performance (i.e., shorter SSRT) in participants with greater preSMA/SMA–STN connectivity and rIFG modulatory activity over preSMA/SMA. Thus, rIFG may be only indirectly connected with M1 and may play a crucial role in modulating preSMA/SMA connectivity. Thus, it is possible to speculate that, relative to the ccPAS study from [43], boosting rIFG to preSMA, our preSMA/SMA to M1 ccPAS protocol can be more efficient in stimulating a more selective connection in action inhibition. Indeed, our previous data [4] demonstrated the crucial role of the rIFG in reactive inhibition, thus, we now question its functional connectivity with M1, not its relevance within the AIN. Importantly, the lack of results in our rIFG‐lM1 group can be ascribed to the ISI employed (i.e., 6 ms). Although our ISI was based on the previous studies, which demonstrated effective connectivity between rIFG and lM1 at this ISI [31, 78], other findings suggested 8 ms to be the ideal ISI for stimulating rIFG–lM1 connectivity [26]. It is important to mention that Picazio and colleagues tested such connectivity during a go/no go task [78], while Lazari and coworkers observed increased CSE selectively 24 h after the ccPAS administration [31]. Thus, more studies are required to investigate further this connection at 8 ms. In conclusion, we successfully demonstrated the efficacy of this ccPAS protocol in improving behavioral inhibition, paving the way for potential clinical application. Indeed, action inhibition impairments are common in clinical and neurodevelopmental conditions such as Parkinsonʼs disease [149, 150, 151, 152, 153, 154], obsessive–compulsive disorder [155], attention deficit hyperactivity disorder [156, 157, 158, 159, 160], and autism [161]. Future studies should verify the feasibility and application of this stimulation protocol on these populations.

## Author Contributions

**Lorenzo Però**: formal analysis, visualization, writing–original draft, writing–review and editing. **Nicolò Arlati**: formal analysis, writing–review and editing. **Laura Lenzi**: data curation, writing–review and editing. **Thomas Quettier**: writing–review and editing. **Simone Battaglia**: methodology, software, visualization, supervision, writing–review and editing. **Sara Borgomaneri**: conceptualization, funding acquisition, methodology, resources, supervision, writing–review and editing.

## Conflicts of Interest

The authors declare no conflicts of interest.

## Data Availability

The data that support the findings of this study are available from the corresponding author upon reasonable request.

## References

[1] R. Zhang , X. Geng , and T. M. C. Lee , “Large‐Scale Functional Neural Network Correlates of Response Inhibition: An fMRI Meta‐Analysis,” Brain Structure and Function 222 (2017): 3973–3990, 10.1007/S00429-017-1443-X. 28551777 PMC5686258

[2] T. Aziz‐Safaie , V. I. Müller , R. Langner , S. B. Eickhoff , and E. C. Cieslik , “The Effect of Task Complexity on the Neural Network for Response Inhibition: An ALE Meta‐Analysis,” Neuroscience and Biobehavioral Reviews 158 (2024): 105544, 10.1016/J.NEUBIOREV.2024.105544. 38220034 PMC11130604

[3] S. Borgomaneri , G. Serio , and S. Battaglia , “Please, Don't Do It! Fifteen Years of Progress of Non‐Invasive Brain Stimulation in Action Inhibition,” Cortex 132 (2020): 404–422, 10.1016/J.CORTEX.2020.09.002. 33045520

[4] S. Battaglia , C. Nazzi , C. Di Fazio , and S. Borgomaneri , “The Role of Pre‐Supplementary Motor Cortex in Action Control With Emotional Stimuli: A Repetitive Transcranial Magnetic Stimulation Study,” Annals of the New York Academy of Science 1536 (2024): 151–166, 10.1111/NYAS.15145. 38751225

[5] A. R. Aron , “From Reactive to Proactive and Selective Control: Developing a Richer Model for Stopping Inappropriate Responses,” Biological Psychiatry 69, no. 12 (2011): e55–e68, 10.1016/J.BIOPSYCH.2010.07.024. 20932513 PMC3039712

[6] T. S. Braver , “The Variable Nature of Cognitive Control: A Dual Mechanisms Framework,” Trends in Cognitive Sciences 16 (2012): 106–113, 10.1016/J.TICS.2011.12.010. 22245618 PMC3289517

[7] H. C. Meyer and D. J. Bucci , “Neural and Behavioral Mechanisms of Proactive and Reactive Inhibition,” Learning and Memory 23 (2016): 504–514, 10.1101/LM.040501.115. 27634142 PMC5026209

[8] G. D. Logan , W. B. Cowan , and K. A. Davis , “On the Ability to Inhibit Simple and Choice Reaction Time Responses: A Model and a Method,” Journal of Experimental Psychology: Human Perception and Performance 10 (1984): 276–291, 10.1037/0096-1523.10.2.276. 6232345

[9] A. R. Aron , T. W. Robbins , and R. A. Poldrack , “Inhibition and the Right Inferior Frontal Cortex: One Decade On,” Trends in Cognitive Sciences 18 (2014): 177–185, 10.1016/J.TICS.2013.12.003. 24440116

[10] S. Jahfari , L. Waldorp , W. P. M. Van Den Wildenberg , H. S. Scholte , K. R. Ridderinkhof , and B. U. Forstmann , “Effective Connectivity Reveals Important Roles for Both the Hyperdirect (Fronto‐Subthalamic) and the Indirect (Fronto‐Striatal‐Pallidal) Fronto‐Basal Ganglia Pathways During Response Inhibition,” Journal of Neuroscience 31 (2011): 6891–6899, 10.1523/JNEUROSCI.5253-10.2011. 21543619 PMC6632844

[11] C. Mancini , N. Modugno , M. Santilli , et al., “Unilateral Stimulation of Subthalamic Nucleus Does Not Affect Inhibitory Control,” Frontiers in Neurology 9 (2019): 432823, 10.3389/FNEUR.2018.01149. PMC6330317 30666229

[12] G. Mirabella , S. Iaconelli , P. Romanelli , et al., “Deep Brain Stimulation of Subthalamic Nuclei Affects Arm Response Inhibition in Parkinson's Patients,” Cerebral Cortex 22 (2012): 1124–1132, 10.1093/CERCOR/BHR187. 21810782

[13] G. Mirabella , S. Iaconelli , N. Modugno , G. Giannini , F. Lena , and G. Cantore , “Stimulation of Subthalamic Nuclei Restores a Near Normal Planning Strategy in Parkinson's Patients,” PLoS ONE 8 (2013): e62793, 10.1371/JOURNAL.PONE.0062793. 23658775 PMC3643906

[14] W. P. M. Van Den Wildenberg , G. J. M. Van Boxtel , M. W. Van Der Molen , D. A. Bosch , J. D. Speelman , and C. H. M. Brunia , “Stimulation of the Subthalamic Region Facilitates the Selection and Inhibition of Motor Responses in Parkinson's Disease,” Journal of Cognitive Neuroscience 18 (2006): 626–636, 10.1162/JOCN.2006.18.4.626. 16768365

[15] W. P. M. Van Den Wildenberg , N. C. Van Wouwe , K. R. Ridderinkhof , et al., “Deep‐Brain Stimulation of the Subthalamic Nucleus Improves Overriding Motor Actions in Parkinson's Disease,” Behavioural Brain Research 402 (2021): 113124, 10.1016/J.BBR.2021.113124. 33422595

[16] A. Tewari , R. Jog , and M. S. Jog , “The Striatum and Subthalamic Nucleus as Independent and Collaborative Structures in Motor Control,” Frontiers in Systems Neuroscience 10 (2016): 17, 10.3389/FNSYS.2016.00017. 26973474 PMC4771745

[17] M. Mattia , P. Pani , G. Mirabella , S. Costa , P. Del Giudice , and S. Ferraina , “Heterogeneous Attractor Cell Assemblies for Motor Planning in Premotor Cortex,” Journal of Neuroscience 33 (2013): 11155–11168, 10.1523/JNEUROSCI.4664-12.2013. 23825419 PMC3718385

[18] G. Mirabella , “Should I Stay or Should I Go? Conceptual Underpinnings of Goal‐Directed Actions,” Frontiers in Systems Neuroscience 8 (2014): 206, 10.3389/FNSYS.2014.00206. 25404898 PMC4217496

[19] G. Mirabella , P. Pani , and S. Ferraina , “Neural Correlates of Cognitive Control of Reaching Movements in the Dorsal Premotor Cortex of Rhesus Monkeys,” Journal of Neurophysiology 106 (2011): 1454–1466, 10.1152/JN.00995.2010. 21697448

[20] C. M. Stinear , J. P. Coxon , and W. D. Byblow , “Primary Motor Cortex and Movement Prevention: Where Stop Meets Go,” Neuroscience and Biobehavioral Reviews 33 (2009): 662–673, 10.1016/J.NEUBIOREV.2008.08.013. 18789963

[21] J. P. Coxon , C. M. Stinear , and W. D. Byblow , “Intracortical Inhibition During Volitional Inhibition of Prepared Action,” Journal of Neurophysiology 95 (2006): 3371–3383, 10.1152/JN.01334.2005. 16495356

[22] P. Di Luzio , L. Brady , S. Turrini , V. Romei , A. Avenanti , and A. Sel , “Investigating the Effects of Cortico‐Cortical Paired Associative Stimulation in the Human Brain: A Systematic Review and Meta‐Analysis,” Neuroscience and Biobehavioral Reviews 167 (2024): 105933, 10.1016/J.NEUBIOREV.2024.105933. 39481669

[23] J. C. Hernandez‐Pavon , A. San Agustín , M. C. Wang , D. Veniero , and J. L. Pons , “Can We Manipulate Brain Connectivity? A Systematic Review of Cortico‐Cortical Paired Associative Stimulation Effects,” Clinical Neurophysiology 154 (2023): 169–193, 10.1016/J.CLINPH.2023.06.016. 37634335

[24] H. Markram , “A History of Spike‐Timing‐Dependent Plasticity,” Frontiers in Synaptic Neuroscience 3 (2011): 1–24, 10.3389/FNSYN.2011.00004. 22007168 PMC3187646

[25] N. Caporale and Y. Dan , “Spike Timing‐Dependent Plasticity: A Hebbian Learning Rule,” Annual Review of Neuroscience 31 (2008): 25–46, 10.1146/ANNUREV.NEURO.31.060407.125639. 18275283

[26] E. R. Buch , V. M. Johnen , N. Nelissen , J. O'Shea , and M. F. S. Rushworth , “Noninvasive Associative Plasticity Induction in a Corticocortical Pathway of the Human Brain,” Journal of Neuroscience 31 (2011): 17669–17679, 10.1523/JNEUROSCI.1513-11.2011. 22131427 PMC6623800

[27] E. Chiappini , S. Borgomaneri , M. Marangon , S. Turrini , V. Romei , and A. Avenanti , “Driving Associative Plasticity in Premotor‐Motor Connections Through a Novel Paired Associative Stimulation Based on Long‐Latency Cortico‐Cortical Interactions,” Brain Stimulation 13 (2020): 1461–1463, 10.1016/j.brs.2020.08.003. 32791314

[28] E. Chiappini , S. Turrini , M. Zanon , M. Marangon , S. Borgomaneri , and A. Avenanti , “Driving Hebbian Plasticity Over Ventral Premotor‐Motor Projections Transiently Enhances Motor Resonance,” Brain Stimulation 17 (2024): 211–220, 10.1016/J.BRS.2024.02.011. 38387557

[29] F. Fiori , E. Chiappini , and A. Avenanti , “Enhanced Action Performance Following TMS Manipulation of Associative Plasticity in Ventral Premotor‐Motor Pathway,” Neuroimage 183 (2018): 847–858, 10.1016/J.NEUROIMAGE.2018.09.002. 30193973

[30] V. M. Johnen , F.‐X. Neubert , E. R. Buch , et al., “Causal Manipulation of Functional Connectivity in a Specific Neural Pathway During Behaviour and at Rest,” Elife 4 (2015): e04585, 10.7554/ELIFE.04585. 25664941 PMC4353194

[31] A. Lazari , P. Salvan , L. Verhagen , et al., “A Macroscopic Link Between Interhemispheric Tract Myelination and Cortico‐Cortical Interactions During Action Reprogramming,” Nature Communications 13 (2022): 1–12, 10.1038/s41467-022-31687-5. PMC9307658 35869067

[32] J. Trajkovic , V. Romei , M. F. S. Rushworth , and A. Sel , “Changing Connectivity Between Premotor and Motor Cortex Changes Inter‐Areal Communication in the Human Brain,” Progress in Neurobiology 228 (2023): 102487, 10.1016/J.PNEUROBIO.2023.102487. 37353108

[33] S. Turrini , F. Fiori , E. Chiappini , E. Santarnecchi , V. Romei , and A. Avenanti , “Gradual Enhancement of Corticomotor Excitability During Cortico‐Cortical Paired Associative Stimulation,” Scientific Reports 12 (2022): 1–8, 10.1038/s41598-022-18774-9. 36038605 PMC9424198

[34] S. Turrini , F. Fiori , N. Bevacqua , et al., “Spike‐Timing‐Dependent Plasticity Induction Reveals Dissociable Supplementary‐ and Premotor‐Motor Pathways to Automatic Imitation,” Proceedings of the National Academy of Sciences 121 (2024): e2404925121, 10.1073/PNAS.2404925121. PMC11228524 38917006

[35] S. Turrini , F. Fiori , E. Chiappini , B. Lucero , E. Santarnecchi , and A. Avenanti , “Cortico‐Cortical Paired Associative Stimulation (ccPAS) Over Premotor‐Motor Areas Affects Local Circuitries in the Human Motor Cortex via Hebbian Plasticity,” Neuroimage 271 (2023): 120027, 10.1016/J.NEUROIMAGE.2023.120027. 36925088

[36] A. Casarotto , E. Dolfini , P. Cardellicchio , L. Fadiga , A. D'Ausilio , and G. Koch , “Mechanisms of Hebbian‐Like Plasticity in the Ventral Premotor–Primary Motor Network,” Journal of Physiology 601 (2023): 211–226, 10.1113/JP283560. 36327142 PMC10100355

[37] A. Casarotto , E. Dolfini , L. Fadiga , G. Koch , and A. D'Ausilio , “Cortico‐Cortical Paired Associative Stimulation Conditioning Superficial Ventral Premotor Cortex–Primary Motor Cortex Connectivity Influences Motor Cortical Activity During Precision Grip,” Journal of Physiology 601 (2023): 3945–3960, 10.1113/JP284500. 37526070

[38] S. Turrini , N. Bevacqua , A. Cataneo , et al., “Neurophysiological Markers of Premotor–Motor Network Plasticity Predict Motor Performance in Young and Older Adults,” Biomedicines 11 (2023): 1464, 10.3390/BIOMEDICINES11051464. 37239135 PMC10216324

[39] S. Turrini , N. Bevacqua , A. Cataneo , et al., “Transcranial Cortico‐Cortical Paired Associative Stimulation (ccPAS) Over Ventral Premotor‐Motor Pathways Enhances Action Performance and Corticomotor Excitability in Young Adults More Than in Elderly Adults,” Frontiers in Aging Neuroscience 15 (2023): 1119508, 10.3389/FNAGI.2023.1119508. 36875707 PMC9978108

[40] N. Bevacqua , S. Turrini , F. Fiori , et al., “Cortico‐Cortical Paired Associative Stimulation Highlights Asymmetrical Communication Between Rostral Premotor Cortices and Primary Motor Cortex,” Brain Stimulation 17 (2024): 89–91, 10.1016/j.brs.2024.01.001. 38191092

[41] E. Chiappini , S. Turrini , F. Fiori , et al., “You Are as Old as the Connectivity You Keep: Distinct Neurophysiological Mechanisms Underlying Age‐Related Changes in Hand Dexterity and Strength,” Archives of Medical Research 56 (2025): 103031, 10.1016/J.ARCMED.2024.103031. 39567344

[42] N. Arai , F. Müller‐Dahlhaus , T. Murakami , et al., “State‐Dependent and Timing‐Dependent Bidirectional Associative Plasticity in the Human SMA‐M1 Network,” Journal of Neuroscience 31 (2011): 15376–15383, 10.1523/JNEUROSCI.2271-11.2011. 22031883 PMC6703519

[43] S. Kohl , R. Hannah , L. Rocchi , C. L. Nord , J. Rothwell , and V. Voon , “Cortical Paired Associative Stimulation Influences Response Inhibition: Cortico‐Cortical and Cortico‐Subcortical Networks,” Biological Psychiatry 85 (2019): 355–363, 10.1016/J.BIOPSYCH.2018.03.009. 29724490 PMC7004814

[44] A. Mandali , K. Tsurumi , T. Popa , and V. Voon , “Replicable Effect of Cortical‐Paired Associative Stimulation on Response Inhibition as a Function of Age,” Brain Stimulation 14 (2021): 788–789, 10.1016/j.brs.2021.04.023. 33965618

[45] S. Battaglia , G. Serio , C. Scarpazza , A. D'Ausilio , and S. Borgomaneri , “Frozen in (E)motion: How Reactive Motor Inhibition Is Influenced by the Emotional Content of Stimuli in Healthy and Psychiatric Populations,” Behaviour Research and Therapy 146 (2021): 103963, 10.1016/J.BRAT.2021.103963. 34530318

[46] L. Pessoa , S. Padmala , A. Kenzer , and A. Bauer , “Interactions Between Cognition and Emotion During Response Inhibition,” Emotion 12 (2012): 192–197, 10.1037/A0024109. 21787074 PMC3208031

[47] H. Kumru , S. Albu , J. Rothwell , et al., “Modulation of Motor Cortex Excitability by Paired Peripheral and Transcranial Magnetic Stimulation,” Clinical Neurophysiology 128 (2017): 2043–2047, 10.1016/J.CLINPH.2017.06.041. 28858700

[48] S. Borgomaneri , F. Vitale , V. Gazzola , and A. Avenanti , “Seeing Fearful Body Language Rapidly Freezes the Observer's Motor Cortex,” Cortex 65 (2015): 232–245, 10.1016/J.CORTEX.2015.01.014. 25835523

[49] F. Faul , E. Erdfelder , A.‐G. Lang , and A. Buchner , “G*Power 3: A Flexible Statistical Power Analysis Program for the Social, Behavioral, and Biomedical Sciences,” Behavior Research Methods 39 (2007): 175–191, 10.3758/BF03193146/METRICS. 17695343

[50] J. F. Veale , “Edinburgh Handedness Inventory—Short Form: A Revised Version Based on Confirmatory Factor Analysis,” Laterality: Asymmetries of Brain, Behaviour, and Cognition 19 (2014): 164–177, 10.1080/1357650X.2013.783045. 23659650

[51] S. Rossi , A. Antal , S. Bestmann , et al., “Safety and Recommendations for TMS Use in Healthy Subjects and Patient Populations, With Updates on Training, Ethical and Regulatory Issues: Expert Guidelines,” Clinical Neurophysiology 132 (2021): 269–306, 10.1016/J.CLINPH.2020.10.003. 33243615 PMC9094636

[52] A. C. Legrand and M. Price , “Emotionally Valenced Stimuli Impact Response Inhibition in Those With Substance Use Disorder and Co‐Occurring Anxiety and Depression Symptoms,” Journal of Affective Disorders 266 (2020): 639–645, 10.1016/J.JAD.2020.02.008. 32056940 PMC7105387

[53] C. D. Spielberger , R. Gorsuch , R. E. Lushene , P. R. Vagg , and G. A. Jacobs , Manual for the State‐Trait Anxiety Inventory (Consulting Psychologists Press, Inc, 1983).

[54] J. H. Patton , M. S. Stanford , and E. S. Barratt , Barratt Impulsiveness Scale‐11 (APA PsycTests, 2011), 10.1037/T05661-000.

[55] J. S. Lappin and C. W. Eriksen , “Use of a Delayed Signal to Stop a Visual Reaction‐Time Response,” Journal of Experimental Psychology 72 (1966): 805–811, 10.1037/H0021266.

[56] G. D. Logan , T. Van Zandt , F. Verbruggen , and E.‐J. Wagenmakers , “On the Ability to Inhibit Thought and Action: General and Special Theories of an Act of Control,” Psychological Review 121 (2014): 66–95, 10.1037/A0035230. 24490789

[57] F. Verbruggen , A. R. Aron , G. P. H Band , et al., “A Consensus Guide to Capturing the Ability to Inhibit Actions and Impulsive Behaviors in the Stop‐Signal Task,” Elife 8 (2019): e46323, 10.7554/ELIFE.46323. 31033438 PMC6533084

[58] S. Battaglia , P. Cardellicchio , C. Di Fazio , C. Nazzi , A. Fracasso , and S. Borgomaneri , “The Influence of Vicarious Fear‐Learning in “Infecting” Reactive Action Inhibition,” Frontiers in Behavioral Neuroscience 16 (2022): 946263, 10.3389/FNBEH.2022.946263. 35941933 PMC9355887

[59] S. Battaglia , P. Cardellicchio , C. Di Fazio , C. Nazzi , A. Fracasso , and S. Borgomaneri , “Stopping in (E)motion: Reactive Action Inhibition When Facing Valence‐Independent Emotional Stimuli,” Frontiers in Behavioral Neuroscience 16 (2022): 998714, 10.3389/FNBEH.2022.998714. 36248028 PMC9561776

[60] S. Borgomaneri , F. Vitale , and A. Avenanti , “Early Changes in Corticospinal Excitability When Seeing Fearful Body Expressions,” Scientific Reports 5 (2015): 1–9, 10.1038/srep14122. PMC4585670 26388400

[61] S. Borgomaneri , V. Gazzola , and A. Avenanti , “Transcranial Magnetic Stimulation Reveals Two Functionally Distinct Stages of Motor Cortex Involvement During Perception of Emotional Body Language,” Brain Structure and Function 220 (2014): 2765–2781, 10.1007/S00429-014-0825-6. 25023734 PMC4549387

[62] S. Borgomaneri , F. Vitale , and A. Avenanti , “Behavioral Inhibition System Sensitivity Enhances Motor Cortex Suppression When Watching Fearful Body Expressions,” Brain Structure and Function 222 (2017): 3267–3282, 10.1007/S00429-017-1403-5. 28357586

[63] S. Borgomaneri , F. Vitale , S. Battaglia , M. De Vega , and A. Avenanti , “Task‐Related Modulation of Motor Response to Emotional Bodies: A TMS Motor‐Evoked Potential Study,” Cortex 171 (2024): 235–246, 10.1016/J.CORTEX.2023.10.013. 38096756

[64] S. Borgomaneri , F. Vitale , and A. Avenanti , “Early Motor Reactivity to Observed Human Body Postures Is Affected by Body Expression, Not Gender,” Neuropsychologia 146 (2020): 107541, 10.1016/J.NEUROPSYCHOLOGIA.2020.107541. 32593723

[65] A. Botta , E. Pelosin , G. Lagravinese , et al., “Modulation of Response Times in Early‐Stage Parkinson's Disease During Emotional Processing of Embodied and Non‐Embodied Stimuli,” Scientific Reports 14 (2024): 1–9, 10.1038/s41598-024-63701-9. 38844758 PMC11156934

[66] A. Botta , G. Lagravinese , M. Bove , A. Avenanti , and L. Avanzino , “Modulation of Response Times During Processing of Emotional Body Language,” Frontiers in Psychology 12 (2021): 616995, 10.3389/FPSYG.2021.616995. 33716882 PMC7947862

[67] A. Botta , G. Lagravinese , M. Bove , et al., “Sensorimotor Inhibition During Emotional Processing,” Scientific Reports 12 (2022): 1–13, 10.1038/s41598-022-10981-8. 35488018 PMC9054825

[68] A. Botta , M. Zhao , J. Samogin , et al., “Early Modulations of Neural Oscillations During the Processing of Emotional Body Language,” Psychophysiology 61 (2024): e14436, 10.1111/PSYP.14436. 37681463

[69] M. Gandolfo , G. D'Argenio , P. E. Downing , and C. Urgesi , “Boosting Forward Connectivity Between Primary Visual and Body Selective Cortex Reduces Interference Between Sex and Emotion Judgements of Bodies,” Neuroimage 310 (2025): 121102, 10.1016/J.NEUROIMAGE.2025.121102. 40081159

[70] V. Oldrati , A. Bardoni , G. Poggi , and C. Urgesi , “Influence of Attention Control on Implicit and Explicit Emotion Processing of Face and Body: Evidence From Flanker and Same‐or‐different Paradigms,” Frontiers in Psychology 10 (2020): 2971, 10.3389/FPSYG.2019.02971. 32038372 PMC6985560

[71] G. D. Logan , R. J. Schachar , and R. Tannock , “Impulsivity and Inhibitory Control,” Psychological Science 8 (1997): 60–64, 10.1111/J.1467-9280.1997.TB00545.X.

[72] M. Senderecka , “Threatening Visual Stimuli Influence Response Inhibition and Error Monitoring: An Event‐Related Potential Study,” Biological Psychology 113 (2016): 24–36, 10.1016/J.BIOPSYCHO.2015.11.003. 26599814

[73] P. M. Hilt , P. Cardellicchio , and A. D'Ausilio , “The Neurophysiology of Action Perception,” in Modelling Human Motion: From Human Perception to Robot Design, ed. N. Noceti , A. Sciutti , and F. Rea (Springer, 2020), 17–32, 10.1007/978-3-030-46732-6_2.

[74] G. P. H. Band , M. W. van der Molen , and G. D. Logan , “Horse‐Race Model Simulations of the Stop‐Signal Procedure,” Acta Psychologica 112 (2003): 105–142, 10.1016/S0001-6918(02)00079-3. 12521663

[75] R. B. Mars , M. C. Klein , F. X. Neubert , et al., “Pre‐SMA Exerts Top‐Down Control Over Primary Motor Cortex During Response Switching: A Paired‐Pulse Transcranial Magnetic Stimulation and Diffusion‐Weighted Imaging Study,” Neuroimage 47 (2009): S177, 10.1016/S1053-8119(09)71932-9.

[76] C. Civardi , R. Cantello , P. Asselman , and J. C. Rothwell , “Transcranial Magnetic Stimulation Can be Used to Test Connections to Primary Motor Areas From Frontal and Medial Cortex in Humans,” Neuroimage 14 (2001): 1444–1453, 10.1006/NIMG.2001.0918. 11707100

[77] W. D. Byblow , J. P. Coxon , C. M. Stinear , et al., “Functional Connectivity Between Secondary and Primary Motor Areas Underlying Hand‐Foot Coordination,” Journal of Neurophysiology 98 (2007): 414–422, 10.1152/JN.00325.2007. 17507503

[78] S. Picazio , D. Veniero , V. Ponzo , et al., “Prefrontal Control Over Motor Cortex Cycles at Beta Frequency During Movement Inhibition,” Current Biology 24 (2014): 2940–2945, 10.1016/j.cub.2014.10.043. 25484293 PMC4274313

[79] S. Van Malderen , M. Hehl , S. Verstraelen , S. P. Swinnen , and K. Cuypers , “Dual‐Site TMS as a Tool to Probe Effective Interactions Within the Motor Network: A Review,” Reviews in the Neurosciences 34 (2022): 129–221, 10.1515/REVNEURO-2022-0020. 36065080

[80] V. Rizzo , H. S. Siebner , F. Morgante , C. Mastroeni , P. Girlanda , and A. Quartarone , “Paired Associative Stimulation of Left and Right Human Motor Cortex Shapes Interhemispheric Motor Inhibition Based on a Hebbian Mechanism,” Cerebral Cortex 19 (2009): 907–915, 10.1093/CERCOR/BHN144. 18791179

[81] V. Rizzo , M. Bove , A. Naro , et al., “Associative Cortico‐Cortical Plasticity May Affect Ipsilateral Finger Opposition Movements,” Behavioural Brain Research 216 (2011): 433–439, 10.1016/J.BBR.2010.08.037. 20816702

[82] S. Koganemaru , T. Mima , M. Nakatsuka , Y. Ueki , H. Fukuyama , and K. Domen , “Human Motor Associative Plasticity Induced by Paired Bihemispheric Stimulation,” Journal of Physiology 587 (2009): 4629–4644, 10.1113/JPHYSIOL.2009.174342. 19687124 PMC2768018

[83] D. Veniero , V. Ponzo , and G. Koch , “Paired Associative Stimulation Enforces the Communication Between Interconnected Areas,” Journal of Neuroscience 33 (2013): 13773–13783, 10.1523/JNEUROSCI.1777-13.2013. 23966698 PMC6618662

[84] G. Koch , V. Ponzo , F. Di Lorenzo , C. Caltagirone , and D. Veniero , “Hebbian and Anti‐Hebbian Spike‐Timing‐Dependent Plasticity of Human Cortico‐Cortical Connections,” Journal of Neuroscience 33 (2013): 9725–9733, 10.1523/JNEUROSCI.4988-12.2013. 23739969 PMC6619701

[85] R. Breveglieri , S. Borgomaneri , M. Filippini , et al., “Complementary Contribution of the Medial and Lateral Human Parietal Cortex to Grasping: A Repetitive TMS Study,” Cerebral Cortex 33 (2023): 5122–5134, 10.1093/CERCOR/BHAC404. 36245221 PMC10152058

[86] R. Breveglieri , S. Borgomaneri , A. Bosco , et al., “rTMS Over the Human Medial Parietal Cortex Impairs Online Reaching Corrections,” Brain Structure and Function 229 (2024): 297–310, 10.1007/S00429-023-02735-7. 38141108 PMC10917872

[87] F. Carducci and R. Brusco , “Accuracy of an Individualized MR‐Based Head Model for Navigated Brain Stimulation,” Psychiatry Research: Neuroimaging 203 (2012): 105–108, 10.1016/J.PSCYCHRESNS.2011.12.013. 22892350

[88] E. Tidoni , S. Borgomaneri , G. Di Pellegrino , and A. Avenanti , “Action Simulation Plays a Critical Role in Deceptive Action Recognition,” Journal of Neuroscience 33 (2013): 611–623, 10.1523/JNEUROSCI.2228-11.2013. 23303940 PMC6704902

[89] F. Fiori , E. Chiappini , M. Soriano , et al., “Long‐Latency Modulation of Motor Cortex Excitability by Ipsilateral Posterior Inferior Frontal Gyrus and Pre‐Supplementary Motor Area,” Scientific Reports 6 (2016): 1–11, 10.1038/srep38396. 27929075 PMC5144072

[90] S. Borgomaneri , M. Zanon , P. Di Luzio , et al., “Increasing Associative Plasticity in Temporo‐Occipital Back‐Projections Improves Visual Perception of Emotions,” Nature Communications 14 (2023): 1–15, 10.1038/s41467-023-41058-3. PMC10517146 37737239

[91] P. Sagaspe , S. Schwartz , and P. Vuilleumier , “Fear and Stop: A Role for the Amygdala in Motor Inhibition by Emotional Signals,” Neuroimage 55 (2011): 1825–1835, 10.1016/J.NEUROIMAGE.2011.01.027. 21272655

[92] M. A. Mayka , D. M. Corcos , S. E. Leurgans , and D. E. Vaillancourt , “Three‐Dimensional Locations and Boundaries of Motor and Premotor Cortices as Defined by Functional Brain Imaging: A Meta‐Analysis,” Neuroimage 31 (2006): 1453–1474, 10.1016/J.NEUROIMAGE.2006.02.004. 16571375 PMC2034289

[93] F. Verbruggen and G. D. Logan , “Response Inhibition in the Stop‐Signal Paradigm,” Trends in Cognitive Sciences 12 (2008): 418–424, 10.1016/j.tics.2008.07.005. 18799345 PMC2709177

[94] M. E. Toplak , R. F. West , and K. E. Stanovich , “Practitioner Review: Do Performance‐Based Measures and Ratings of Executive Function Assess the Same Construct?,” Journal of Child Psychology and Psychiatry 54 (2013): 131–143, 10.1111/JCPP.12001. 23057693

[95] J. Shuster and M. E. Toplak , “Executive and Motivational Inhibition: Associations With Self‐Report Measures Related to Inhibition,” Consciousness and Cognition 18 (2009): 471–480, 10.1016/J.CONCOG.2009.01.004. 19233688

[96] R. G. Carson , A. Capozio , E. Mcnickle , and A. T. Sack , “A Bayesian Approach to Analysing Cortico‐Cortical Associative Stimulation Induced Increases in the Excitability of Corticospinal Projections in Humans,” Experimental Brain Research 239 (2021): 21–30, 10.1007/S00221-020-05943-3. 33097986 PMC7884589

[97] P. E. Green , M. C. Ridding , K. D. Hill , J. G. Semmler , P. D. Drummond , and A.‐M. Vallence , “Supplementary Motor Area—Primary Motor Cortex Facilitation in Younger but Not Older Adults,” Neurobiology of Aging 64 (2018): 85–91, 10.1016/J.NEUROBIOLAGING.2017.12.016. 29348045

[98] B. K. Rurak , J. P. Rodrigues , B. D. Power , P. D. Drummond , and A. M. Vallence , “Test Re‐Test Reliability of Dual‐Site TMS Measures of SMA‐M1 Connectivity Differs Across Inter‐Stimulus Intervals in Younger and Older Adults,” Neuroscience 472 (2021): 11–24, 10.1016/J.NEUROSCIENCE.2021.07.023. 34333064

[99] C. Neige , P. Vassiliadis , A. Ali Zazou , et al., “Connecting the Dots: Harnessing Dual‐Site Transcranial Magnetic Stimulation to Quantify the Causal Influence of Medial Frontal Areas on the Motor Cortex,” Cerebral Cortex 33 (2023): 11339–11353, 10.1093/CERCOR/BHAD370. 37804253

[100] H. Tokuno , “Organization of Nonprimary Motor Cortical Inputs on Pyramidal and Nonpyramidal Tract Neurons of Primary Motor Cortex: An Electrophysiological Study in the Macaque Monkey,” Cerebral Cortex 10 (2000): 58–68, 10.1093/CERCOR/10.1.58. 10639396

[101] K. F. Muakkassa and P. L. Strick , “Frontal Lobe Inputs to Primate Motor Cortex: Evidence for Four Somatotopically Organized ‘Premotor’ Areas,” Brain Research 177, no. 1 (1979): 176–182, 10.1016/0006-8993(79)90928-4. 115545

[102] G. Luppino , M. Matelli , R. Camarda , and G. Rizzolatti , “Corticocortical Connections of Area F3 (SMA‐Proper) and Area F6 (Pre‐SMA) in the Macaque Monkey,” Journal of Comparative Neurology 338 (1993): 114–140, 10.1002/CNE.903380109. 7507940

[103] K. Shima and J. Tanji , “Role for Cingulate Motor Area Cells in Voluntary Movement Selection Based on Reward,” Science 282 (1998): 1335–1338, 10.1126/SCIENCE.282.5392.1335. 9812901

[104] M. Isoda and O. Hikosaka , “Role for Subthalamic Nucleus Neurons in Switching From Automatic to Controlled Eye Movement,” Journal of Neuroscience 28 (2008): 7209–7218, 10.1523/JNEUROSCI.0487-08.2008. 18614691 PMC2667154

[105] A. Nambu , H. Tokuno , I. Hamada , et al., “Excitatory Conical Inputs to Pallidal Neurons via the Subthalamic Nucleus in the Monkey,” Journal of Neurophysiology 84 (2000): 289–300, 10.1152/JN.2000.84.1.289. 10899204

[106] M. Ullsperger and D. Y. Von Cramon , “Subprocesses of Performance Monitoring: A Dissociation of Error Processing and Response Competition Revealed by Event‐Related fMRI and ERPs,” Neuroimage 14 (2001): 1387–1401, 10.1006/NIMG.2001.0935. 11707094

[107] K. Shima , H. Mushiake , N. Saito , and J. Tanji , “Role for Cells in the Presupplementary Motor Area in Updating Motor Plans,” Proceedings of the National Academy of Sciences 93 (1996): 8694–8698, 10.1073/PNAS.93.16.8694. PMC38735 8710933

[108] M. Isoda and O. Hikosaka , “Switching From Automatic to Controlled Action by Monkey Medial Frontal Cortex,” Nature Neuroscience 10 (2007): 240–248, 10.1038/nn1830. 17237780

[109] J. Chikazoe , K. Jimura , S. Hirose , K.‐I. Yamashita , Y. Miyashita , and S. Konishi , “Preparation to Inhibit a Response Complements Response Inhibition During Performance of a Stop‐Signal Task,” Journal of Neuroscience 29 (2009): 15870–15877, 10.1523/JNEUROSCI.3645-09.2009. 20016103 PMC6666181

[110] S. Jahfari , C. M. Stinear , M. Claffey , F. Verbruggen , and A. R. Aron , “Responding With Restraint: What Are the Neurocognitive Mechanisms?,” Journal of Cognitive Neuroscience 22 (2010): 1479–1492, 10.1162/JOCN.2009.21307. 19583473 PMC2952035

[111] H. W. Lee , M.‐S. Lu , C.‐Y. Chen , N. G. Muggleton , T.‐Y. Hsu , and C.‐H. Juan , “Roles of the Pre‐SMA and rIFG in Conditional Stopping Revealed by Transcranial Magnetic Stimulation,” Behavioural Brain Research 296 (2016): 459–467, 10.1016/J.BBR.2015.08.024. 26304720

[112] W. Cai , C. L. Oldenkamp , and A. R. Aron , “Stopping Speech Suppresses the Task‐Irrelevant Hand,” Brain and Language 120 (2012): 412–415, 10.1016/J.BANDL.2011.11.006. 22206872 PMC3533487

[113] I. Obeso , L. Wilkinson , J. T. Teo , P. Talelli , J. C. Rothwell , and M. Jahanshahi , “Theta Burst Magnetic Stimulation Over the Pre‐Supplementary Motor Area Improves Motor Inhibition,” Brain Stimulation 10 (2017): 944–951, 10.1016/J.BRS.2017.05.008. 28624346

[114] A. D. Bender , H. L. Filmer , and P. E. Dux , “Transcranial Direct Current Stimulation of Superior Medial Frontal Cortex Disrupts Response Selection During Proactive Response Inhibition,” Neuroimage 158 (2017): 455–465, 10.1016/J.NEUROIMAGE.2016.10.035. 27789261

[115] C. S. R. Li , C. Huang , R. T. Constable , et al., “Imaging Response Inhibition in a Stop‐Signal Task: Neural Correlates Independent of Signal Monitoring and Post‐Response Processing,” Journal of Neuroscience 26 (2006): 186–192, 10.1523/JNEUROSCI.3741-05.2006. 16399686 PMC6674298

[116] D. Floden and D. T. Stuss , “Inhibitory Control Is Slowed in Patients With Right Superior Medial Frontal Damage,” Journal of Cognitive Neuroscience 18 (2006): 1843–1849, 10.1162/JOCN.2006.18.11.1843. 17069475

[117] S. H. Mostofsky and D. J. Simmonds , “Response Inhibition and Response Selection: Two Sides of the Same Coin,” Journal of Cognitive Neuroscience 20 (2008): 751–761, 10.1162/JOCN.2008.20500. 18201122

[118] P. C. J. Taylor , A. C. Nobre , and M. F. S. Rushworth , “Subsecond Changes in Top–Down Control Exerted by Human Medial Frontal Cortex During Conflict and Action Selection: A Combined Transcranial Magnetic Stimulation–Electroencephalography Study,” Journal of Neuroscience 27 (2007): 11343–11353, 10.1523/JNEUROSCI.2877-07.2007. 17942729 PMC6673042

[119] D. S. A. Majid , W. Cai , J. Corey‐Bloom , and A. R. Aron , “Proactive Selective Response Suppression Is Implemented via the Basal Ganglia,” Journal of Neuroscience 33 (2013): 13259–13269, 10.1523/JNEUROSCI.5651-12.2013. 23946385 PMC3742918

[120] M. Oliveri , C. Babiloni , M. M. Filippi , et al., “Influence of the Supplementary Motor Area on Primary Motor Cortex Excitability During Movements Triggered by Neutral or Emotionally Unpleasant Visual Cues,” Experimental Brain Research 149 (2003): 214–221, 10.1007/S00221-002-1346-8. 12610690

[121] F. Di Gregorio and S. Battaglia , “The Intricate Brain–Body Interaction in Psychiatric and Neurological Diseases,” Advances in Clinical and Experimental Medicine 33 (2024): 321–326, 10.17219/ACEM/185689. 38515256

[122] S. Battaglia , C. D. Fazio , S. Borgomaneri , and A. Avenanti , “Cortisol Imbalance and Fear Learning in PTSD: Therapeutic Approaches to Control Abnormal Fear Responses,” Current Neuropharmacology 23 (2025): 835–846, 10.2174/1570159x23666250123142526. 39851130 PMC12163469

[123] A. R. Aron , D. M. Herz , P. Brown , B. U. Forstmann , and K. Zaghloul , “Frontosubthalamic Circuits for Control of Action and Cognition,” Journal of Neuroscience 36 (2016): 11489–11495, 10.1523/JNEUROSCI.2348-16.2016. 27911752 PMC5125216

[124] S. Battaglia , C. Di Fazio , M. Mazzà , M. Tamietto , and A. Avenanti , “Targeting Human Glucocorticoid Receptors in Fear Learning: A Multiscale Integrated Approach to Study Functional Connectivity,” International Journal of Molecular Sciences 25 (2024): 864, 10.3390/IJMS25020864. 38255937 PMC10815285

[125] N. M. Drummond and R. Chen , “Deep Brain Stimulation and Recordings: Insights Into the Contributions of Subthalamic Nucleus in Cognition,” Neuroimage 222 (2020): 117300, 10.1016/J.NEUROIMAGE.2020.117300. 32828919

[126] L. Mallet , M. Schüpbach , K. N'Diaye , et al., “Stimulation of Subterritories of the Subthalamic Nucleus Reveals Its Role in the Integration of the Emotional and Motor Aspects of Behavior,” Proceedings of the National Academy of Sciences 104 (2007): 10661–10666, 10.1073/PNAS.0610849104. PMC1965569 17556546

[127] C. Nazzi , A. Avenanti , and S. Battaglia , “The Involvement of Antioxidants in Cognitive Decline and Neurodegeneration: *Mens sana in corpore sano* ,” Antioxidants 13 (2024): 701, 10.3390/ANTIOX13060701. 38929140 PMC11200558

[128] T. Sieger , T. Serranová , F. Růžička , et al., “Distinct Populations of Neurons Respond to Emotional Valence and Arousal in the Human Subthalamic Nucleus,” Proceedings of the National Academy of Sciences 112 (2015): 3116–3121, 10.1073/PNAS.1410709112. PMC4364224 25713375

[129] S. Jahfari , F. Verbruggen , M. J. Frank , et al., “How Preparation Changes the Need for Top–Down Control of the Basal Ganglia When Inhibiting Premature Actions,” Journal of Neuroscience 32 (2012): 10870–10878, 10.1523/JNEUROSCI.0902-12.2012. 22875921 PMC6621019

[130] P. Nachev , H. Wydell , K. O'Neill , M. Husain , and C. Kennard , “The Role of the Pre‐Supplementary Motor Area in the Control of Action,” Neuroimage 36 (2007): T155–T163, 10.1016/J.NEUROIMAGE.2007.03.034. 17499162 PMC2648723

[131] K. Shima and J. Tanji , “Both Supplementary and Presupplementary Motor Areas Are Crucial for the Temporal Organization of Multiple Movements,” Journal of Neurophysiology 80 (1998): 3247–3260, 10.1152/JN.1998.80.6.3247. 9862919

[132] S. E. Williams , E. J. Lenze , and J. D. Waring , “Positive Information Facilitates Response Inhibition in Older Adults Only When Emotion Is Task‐Relevant,” Cognition and Emotion 34 (2020): 1632–1645, 10.1080/02699931.2020.1793303. 32677540 PMC7677201

[133] C. Mancini , L. Falciati , C. Maioli , and G. Mirabella , “Happy Facial Expressions Impair Inhibitory Control With Respect to Fearful Facial Expressions but Only When Task‐Relevant,” Emotion 22 (2022): 142–152, 10.1037/EMO0001058. 34968143

[134] M. Calbi , M. Montalti , C. Pederzani , et al., “Emotional Body Postures Affect Inhibitory Control Only When Task‐Relevant,” Frontiers in Psychology 13 (2022): 1035328, 10.3389/FPSYG.2022.1035328. 36405118 PMC9669573

[135] M. Senderecka , “Emotional Enhancement of Error Detection—The Role of Perceptual Processing and Inhibition Monitoring in Failed Auditory Stop Trials,” Cognitive, Affective, & Behavioral Neuroscience 18 (2017): 1–20, 10.3758/S13415-017-0546-4. PMC5823965 29076064

[136] F. Moscatelli , G. Messina , A. Valenzano , et al., “Functional Assessment of Corticospinal System Excitability in Karate Athletes,” PLoS ONE 11 (2016): e0155998, 10.1371/JOURNAL.PONE.0155998. 27218465 PMC4878742

[137] F. Moscatelli , G. Messina , A. Valenzano , et al., “Differences in Corticospinal System Activity and Reaction Response Between Karate Athletes and Non‐Athletes,” Neurological Sciences 37 (2016): 1947–1953, 10.1007/S10072-016-2693-8. 27544220

[138] J. F. M. Müller‐Dahlhaus , Y. Orekhov , Y. Liu , and U. Ziemann , “Interindividual Variability and Age‐Dependency of Motor Cortical Plasticity Induced by Paired Associative Stimulation,” Experimental Brain Research 187 (2008): 467–475, 10.1007/S00221-008-1319-7. 18320180

[139] A. Hampshire and D. J. Sharp , “Contrasting Network and Modular Perspectives on Inhibitory Control,” Trends in Cognitive Sciences 19 (2015): 445–452, 10.1016/J.TICS.2015.06.006. 26160027

[140] A. Hampshire , S. R. Chamberlain , M. M. Monti , J. Duncan , and A. M. Owen , “The Role of the Right Inferior Frontal Gyrus: Inhibition and Attentional Control,” Neuroimage 50 (2010): 1313–1319, 10.1016/J.NEUROIMAGE.2009.12.109. 20056157 PMC2845804

[141] M. Erika‐Florence , R. Leech , and A. Hampshire , “A Functional Network Perspective on Response Inhibition and Attentional Control,” Nature Communications 5 (2014): 1–12, 10.1038/ncomms5073. PMC4059922 24905116

[142] C. C. Yang , N. Khalifa , and B. Völlm , “Excitatory Repetitive Transcranial Magnetic Stimulation Applied to the Right Inferior Frontal Gyrus Has no Effect on Motor or Cognitive Impulsivity in Healthy Adults,” Behavioural Brain Research 347 (2018): 1–7, 10.1016/J.BBR.2018.02.047. 29505803

[143] A. R. Aron , P. C. Fletcher , E. T. Bullmore , B. J. Sahakian , and T. W. Robbins , “Stop‐Signal Inhibition Disrupted by Damage to Right Inferior Frontal Gyrus in Humans,” Nature Neuroscience 6 (2003): 115–116, 10.1038/nn1003. 12536210

[144] A. R. Aron and R. A. Poldrack , “Cortical and Subcortical Contributions to Stop Signal Response Inhibition: Role of the Subthalamic Nucleus,” Journal of Neuroscience 26 (2006): 2424–2433, 10.1523/JNEUROSCI.4682-05.2006. 16510720 PMC6793670

[145] F.‐X. Neubert , R. B. Mars , E. R. Buch , E. Olivier , and M. F. S. Rushworth , “Cortical and Subcortical Interactions During Action Reprogramming and Their Related White Matter Pathways,” Proceedings of the National Academy of Sciences 107 (2010): 13240–13245, 10.1073/PNAS.1000674107. PMC2922153 20622155

[146] P. Nachev , C. Kennard , and M. Husain , “Functional Role of the Supplementary and Pre‐Supplementary Motor Areas,” Nature Reviews Neuroscience 9 (2008): 856–869, 10.1038/nrn2478. 18843271

[147] R. Mukamel , A. D. Ekstrom , J. Kaplan , M. Iacoboni , and I. Fried , “Single‐Neuron Responses in Humans During Execution and Observation of Actions,” Current Biology 20 (2010): 750–756, 10.1016/j.cub.2010.02.045. 20381353 PMC2904852

[148] C. L. Rae , L. E. Hughes , M. C. Anderson , and J. B. Rowe , “The Prefrontal Cortex Achieves Inhibitory Control by Facilitating Subcortical Motor Pathway Connectivity,” Journal of Neuroscience 35 (2015): 786–794, 10.1523/JNEUROSCI.3093-13.2015. 25589771 PMC4293423

[149] S. Battaglia , A. Avenanti , L. Vécsei , and M. Tanaka , “Neurodegeneration in Cognitive Impairment and Mood Disorders for Experimental, Clinical and Translational Neuropsychiatry,” Biomedicines 12 (2024): 574, 10.3390/BIOMEDICINES12030574. 38540187 PMC10968650

[150] M. Tanaka , S. Battaglia , and D. Liloia , “Navigating Neurodegeneration: Integrating Biomarkers, Neuroinflammation, and Imaging in Parkinson's, Alzheimer's, and Motor Neuron Disorders,” Biomedicines 13 (2025): 1045, 10.3390/BIOMEDICINES13051045. 40426872 PMC12109388

[151] G. Mirabella , A. Pilotto , A. Rizzardi , et al., “Effects of Dopaminergic Treatment on Inhibitory Control Differ Across Hoehn and Yahr Stages of Parkinson's Disease,” Brain Communications 6 (2023): fcad350, 10.1093/BRAINCOMMS/FCAD350. 38162902 PMC10757450

[152] M. Tanaka , S. Battaglia , L. Giménez‐Llort , et al., “Innovation at the Intersection: Emerging Translational Research in Neurology and Psychiatry,” Cells 13 (2024): 790, 10.3390/CELLS13100790. 38786014 PMC11120114

[153] S. Gauggel , M. Rieger , and T. A. Feghoff , “Inhibition of Ongoing Responses in Patients With Parkinson's Disease,” Journal of Neurology, Neurosurgery, and Psychiatry 75 (2004): 539–544, 10.1136/JNNP.2003.016469. 15026491 PMC1739013

[154] S. A. Wylie , W. P. M. Van Den Wildenberg , K. R. Ridderinkhof , et al., “The Effect of Parkinson's Disease on Interference Control During Action Selection,” Neuropsychologia 47 (2009): 145–157, 10.1016/J.NEUROPSYCHOLOGIA.2008.08.001. 18761363 PMC4524676

[155] R. Penadés , R. Catalán , K. Rubia , et al., “Impaired Response Inhibition in Obsessive Compulsive Disorder,” European Psychiatry 22 (2007): 404–410, 10.1016/J.EURPSY.2006.05.001. 17127038

[156] A. R. Aron and R. A. Poldrack , “The Cognitive Neuroscience of Response Inhibition: Relevance for Genetic Research in Attention‐Deficit/Hyperactivity Disorder,” Biological Psychiatry 57 (2005): 1285–1292, 10.1016/J.BIOPSYCH.2004.10.026. 15950000

[157] J. T. Nigg , G. Stavro , M. Ettenhofer , D. Z. Hambrick , T. Miller , and J. M. Henderson , “Executive Functions and ADHD in Adults: Evidence for Selective Effects on ADHD Symptom Domains,” Journal of Abnormal Psychology 114 (2005): 706–717, 10.1037/0021-843X.114.3.706. 16351391

[158] K. R. Ridderinkhof , A. Scheres , J. Oosterlaan , and J. A. Sergeant , “Delta Plots in the Study of Individual Differences: New Tools Reveal Response Inhibition Deficits in AD/HD That Are Eliminated by Methylphenidate Treatment,” Journal of Abnormal Psychology 114 (2005): 197–215, 10.1037/0021-843X.114.2.197. 15869351

[159] B. M. Van Hulst , P. De Zeeuw , C. Vlaskamp , Y. Rijks , B. B. Zandbelt , and S. Durston , “Children With ADHD Symptoms Show Deficits in Reactive but Not Proactive Inhibition, Irrespective of Their Formal Diagnosis,” Psychological Medicine 48 (2018): 2515–2521, 10.1017/S0033291718000107. 29415788 PMC6190063

[160] A. Chevrier and R. J. Schachar , “BOLD Differences Normally Attributed to Inhibitory Control Predict Symptoms, Not Task‐Directed Inhibitory Control in ADHD,” Journal of Neurodevelopmental Disorders 12 (2020): 1–12, 10.1186/S11689-020-09311-8. 32085698 PMC7035717

[161] L. M. Schmitt , S. P. White , E. H. Cook , J. A. Sweeney , and M. W. Mosconi , “Cognitive Mechanisms of Inhibitory Control Deficits in Autism Spectrum Disorder,” Journal of Child Psychology and Psychiatry 59 (2018): 586–595, 10.1111/JCPP.12837. 29052841 PMC5906199

